# SMYD2‐mediated TRAF2 methylation promotes the NF‐κB signaling pathways in inflammatory diseases

**DOI:** 10.1002/ctm2.591

**Published:** 2021-11-04

**Authors:** Weijun Wu, Jinghuan Wang, Chenxi Xiao, Zhenghua Su, Haibi Su, Wen Zhong, Jianchun Mao, Xinhua Liu, Yi Zhun Zhu

**Affiliations:** ^1^ School of Pharmacy Human Phenome Institute Fudan University Shanghai 201203 China; ^2^ State Key Laboratory of Quality Research in Chinese Medicine and School of Pharmacy Macau University of Science and Technology Macau China; ^3^ Department of Rhumatology Shanghai Longhua Hospital Shanghai University of Traditional Chinese Medicine Shanghai 201203 China

**Keywords:** inflammatory diseases, NF‐κB, SMYD2, TRAF2

## Abstract

**Background:**

The methylation of lysine residues has been involved in the multiple biological and diseases processes. Recently, some particular non‐histone proteins have been elucidated to be methylated by SMYD2, a SET and MYND domain protein with lysine methyltransferase activity.

**Methods:**

SMYD2 was evaluated in synovial tissue and cells derived from rheumatoid arthritis patients. We confirmed TRAF2 could be methylated by SMYD2 using Mass spectrometry, pull‐down, immunoprecipitation, methyltransferase assay, ubiquitination assay, luciferase reporter assays, and western blot analyses. Using loss‐ and gain‐of function studies, we explored the biological functions of SMYD2 in vitro and in vivo. Using acute and chronic inflammation with different mice models to determine the impact of SMYD2.

**Results:**

Here, we first time confirmed that the cytoplasmic protein TRAF2 as the kernel node for NF‐κB signaling pathway could be methylated by SMYD2. SMYD2‐mediated TRAF2 methylation contributed to the durative sensitization of NF‐κB signaling transduction through restraining its own proteolysis and enhancing the activity. In addition, we found knocking down of SMYD2 has different degrees of mitigation in acute and chronic inflammation mice models. Furthermore, as the lysine‐specific demethylase, LSD1 could resist methylation on TRAF2 induced by SMYD2.

**Conclusions:**

Our data uncovered an unprecedented cytoplasmic protein network that employed methylation of TRAF2 for the maintenance of NF‐κB activation during inflammatory diseases.

## Graphical abstract

We confirmed that the cytoplasmic protein TRAF2 as the kernel node for the activation of the NF‐κB signaling pathway could be methylated by SMYD2 in various inflammatory diseases, and methylation of TRAF2 by SMYD2 contributed to the durative sensitization of NF‐κB signaling transduction through restraining its proteolysis and enhancing the activity.

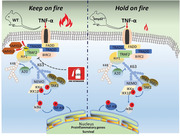

## 1 BACKGROUND

SMYD2 was first confirmed as one of the 5‐member SET and MYND domain (SMYD) family of proteins to methylate histone H3K36 and H3K4 with the SET‐dependent manner.^1^, ^2^ It also was known to methylate the non‐histone protein, including the tumor suppressive factor p53, RB1 and carcinogen HSP90.^3^, ^4^, ^5^ The majority of its substrates play the crucial roles in tumor‐associated processes. Not limited to the oncology, it was illustrated that the elevation of SMYD2 could further methylate NF‐κB and STAT3 to enhance their phosphorylation and which forms the positive feedback loop to induce the renal cyst growth and the inflammation in kidney disease.^6^ However, the previous report demonstrated that SMYD2 as the negative regulator mediated H3K36 dimethylation at *Tnf* and *Ii‐6* promoters to suppress the activation of macrophage during inflammation.^7^ So, the effects of SMYD2 on inflammation is still contradictory and poorly understood, typically in the innate immune system.

NF‐κB has been defined as the “holy grail” for a target for developing a brand‐new anti‐inflammatory drug. NF‐κB plays the pivotal role in the survival, activation and differentiation of various innate immune cells that potentially functions the first line of defense against the foreign invasion.^8^, ^9^ In addition, NF‐κB induces the expression of multiple pro‐inflammatory genes encoding chemokines and cytokines, and is a critical mediator of an inflammatory response.^10^, ^11^ There are two main different NF‐κB‐activating pathways in cells, the canonical pathway and the noncanonical pathway.^12^ Under the resting conditions, the inhibitory IκB protein binds to NF‐κB dimers to keep inactive NF‐κB complexes in the cytoplasm. The stimulus‐induced phosphorylation of IκB kinase (IKK) triggers proteasomal degradation of IκB. After the degradation of IκB, NF‐κB dimers are released and translocate to the nucleus.^9^ The canonical pathway is often initiated by most physiological NF‐κB stimuli binding to TNFR1. TNFR1 leads to the binding of TRADD to set up the platform for the recruitment of FADD and TRAF2, then TRAF2 interacts with RIPK1 for IKK activation and finally ignite the degradation of IκB to release NF‐κB.^13^ As for the noncanonical NF‐κB pathway, some specific TNF family cytokines including CD40L, lymphotoxin‐β (LT‐β) and B‐cell activating factor of TNF family (BAFF) mediate phosphorylation of p100, which causes partial processing of p100 followed with the formation of transcriptionally active p52 complex.^14^ The process of IKKα activation and phosphorylation of p100 depend on NIK and which is subjected to the complexes composed with TRAF2, TRAF3 and BIRC2.^15^ In addition, NF‐κB activation depends on inducing numerous post‐translational modifications (PTM) of either IκBs, IKKs, p100 and even the TRAF2. It has become more and more clear that various other covalent changes to existing proteins could have an equally vital effect on protein activity, such as the methylation of the proteins on lysine or arginine residues, as well as nitrosylation or acetylation of proteins.

**HIGHLIGHTS**

- SMYD2 is increased in synovial tissue and cells derived from rheumatoid arthritis patients.
- The cytoplasmic protein TRAF2 can be methylated by SMYD2 in various inflammatory diseases.
- TRAF2 methylation contributes to the durative sensitization of NF‐κB signaling transduction.
- Knocking down of SMYD2 has different degrees of mitigation in acute and chronic inflammation mice models.

TRAF family includes seven members in mammalian cells and has been reported to engage in innate and adaptive immunity. All TRAFs share a universal RING domain that potentially functions as E3 ubiquitin ligase.^16^ TNFR1 can bind with the adaptor TRADD, which works as the platform for recruitment of FADD and TRAF2, then TRAF2 combines with ubiquitin ligase BIRC2, BIRC3 and kinase RIPK1. RIPK1 undergoing K63‐linked ubiquitination modification recruits and activates kinase TAK1 and IKK complex. For the noncanonical NF‐κB signaling, TRAF2 could mediate the stabilization of NIK through degradation of its negative regulator TRAF3 with the help of BIRC2/3.^16^

Here, we reveal that TRAF2 is the crucial positive factor for the activation of NF‐κB signaling in inflammation and the methylation of TRAF2 induced by SMYD2 could aggravate the inflammatory response. The methylation of TRAF2 avoids degradation by ubiquitin‐proteasome proteolytic, thus stabilizing TRAF2, which contributes to the prolonged NF‐κB signaling transduction in inflammatory diseases. Meanwhile, the first identified demethylase LSD1 has the capacity of reversing the methylation of TRAF2.

## 2 RESULTS

### 2.1 SMYD2 is anomalous existence in the synovium of rheumatoid arthritis (RA) samples

**FIGURE 1:**
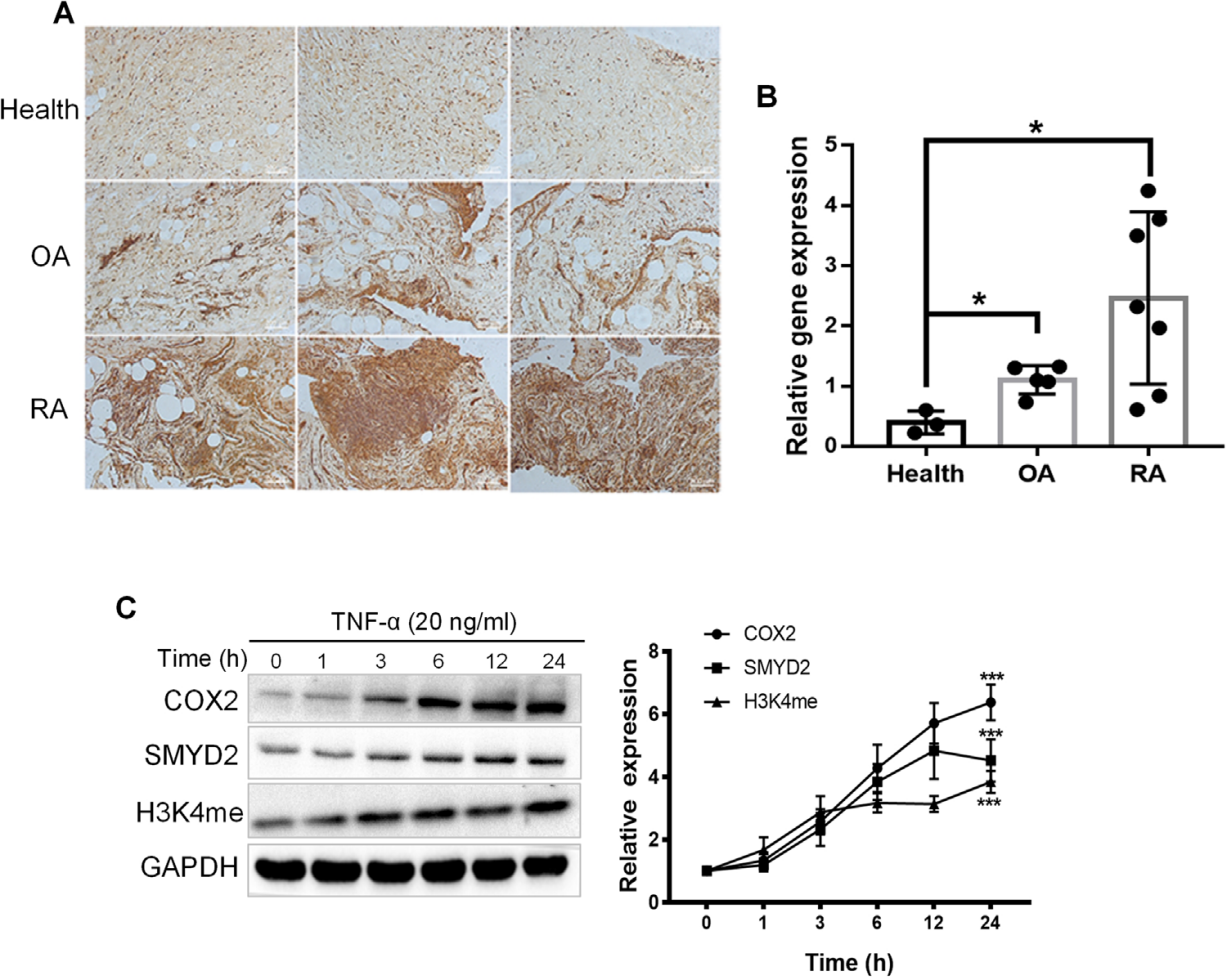
SMYD2 expression is increased in synovial tissue and fibroblast‐like synoviocytes (FLS) from RA patients. Representative images of immunohistochemistry microscopy of SMYD2 in knee joints sections (A) and mRNA expression of SMYD2 in healthy, RA or OA patients’ synovium, data are presented as mean ± S.D; *p < 0.05, n = 3‐7/group (B). (C) SMYD2 expression is increased in TNF‐α‐induced FLS from RA patients. FLS cells were treated with TNF‐α (20 ng/ml) for the indicated times, immunoblot analyzed for SMYD2, COX2, and H3K4me. Data are presented as mean ± S.D, n = 3, ***p < 0.001 vs 0 h, Control = normal human; RA = rheumatoid arthritis; OA = osteoarthritis

Although a part of biologic functions for SMYD2 has been clarified, the roles of SMYD2 in non‐neoplastic cellular biology and diseases like inflammation remain largely unclear. We examined SMYD2 expression in clinical synovium samples collected from RA and osteoarthritis (OA) patients and healthy donators. We found a significant increase of SMYD2 expression in RA compared with healthy or OA tissues (Figure 1A and B). Then we confirmed the significant elevation of SMYD2 expression in primary synovial cells insulted with 20 ng/ml TNF‐α. As one of SMYD2 targeting histone substrate, the monomethylated H3K4 (H3K4me) was also raised with the time‐dependent manner (Figure 1C). Those results indicate that SMYD2 may play a pivotal role in mediating the pathogenesis of RA.

### 2.2 SMYD2 monomethylated H3K4 enhances the expression of A20

A20, known as tumor necrosis factor alpha‐induced protein 3 (*Tnfaip3*), is a deubiquitinase enzyme with a crucial function in the inhibition of various proinflammatory molecules, including RIPK1 and IKKγ. It has been demonstrated that Ash1L, an H3K4 methyltransferase, could enhance A20 expression through induction of H3K4 modification at the *Tnfaip3* promoter.^19^ Considering SMYD2 possessing the SET domain and targeting H3K4, here we speculated that SMYD2 could also enhance the expression of A20. We performed the Chromatin immunoprecipitation (ChIP) experiments with antibody binding to H3K4me. The results indicated that SMYD2 could indeed facilitate A20 expression through its HMTase activity upon TNF‐α stimulation (Figure S1A). Furthermore, we used the siRNA against *SMYD2*, the results revealed that silence of SMYD2 led to the reduced A20 mRNA expression in TNF‐α stimulated cells (Figure S1B). Hence SMYD2 recruits H3K4me to the *Tnfaip3* promoter, to enhance the expression of A20 with the TNF‐α stimulation in synovial cells.

### 2.3 SMYD2 acts as the positive regulator for the NF‐κB signaling cascade

**FIGURE 2:**
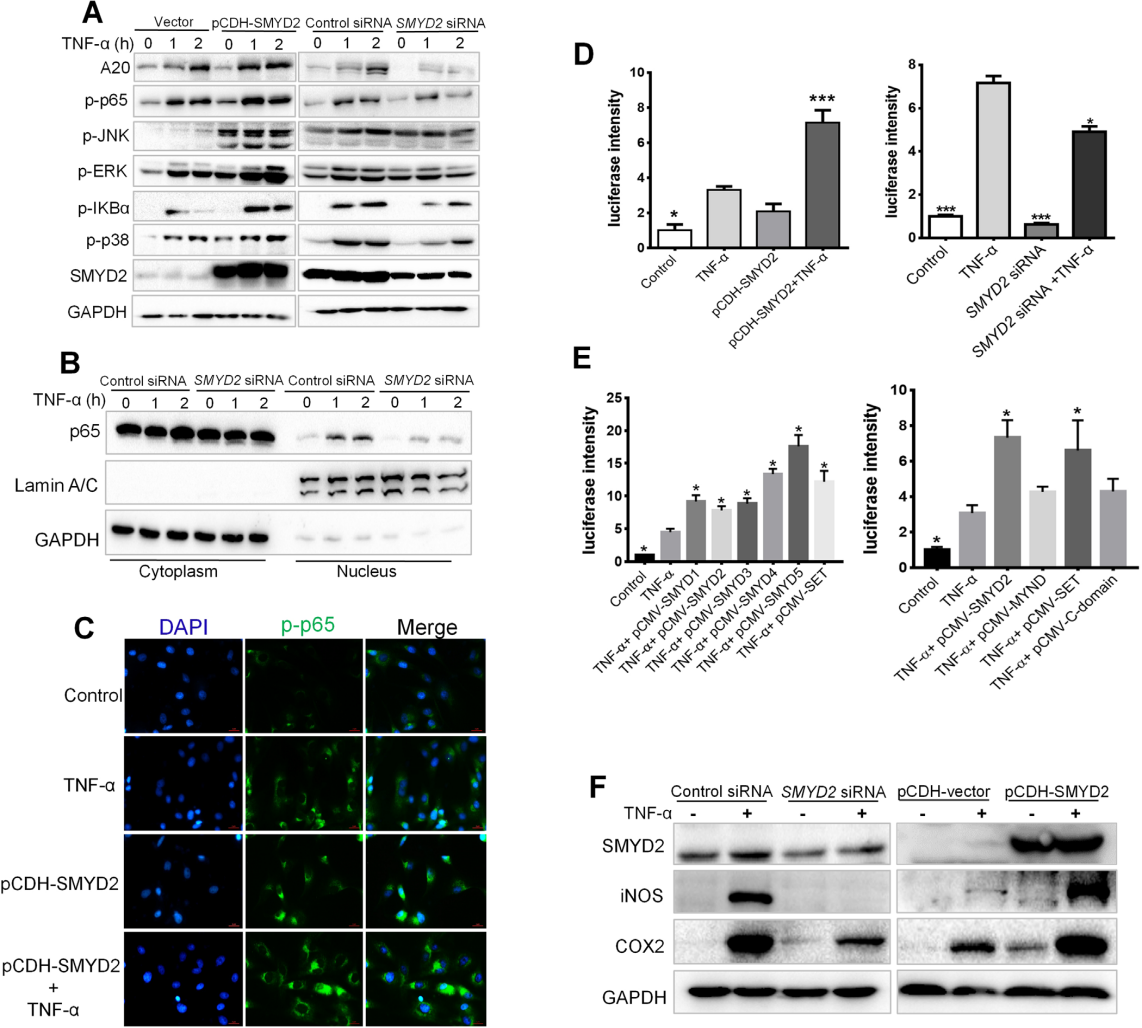
SMYD2 positively regulates the NF‐κB signaling cascade. Fibroblast‐like synoviocytes (FLS) were transfected with SMYD2 siRNA oligos or pCDH‐SMYD2, then treated with TNF‐α for 1 h and 2 h, cell lysates were prepared and blotted with phosphospecific antibodies to p65 and MAPK (A); Nuclear and cytoplasm distribution of p65 were examined by western blot (B); The cells were immunostained with anti‐p‐p65 and analyzed by fluorescence microscopy; scale bars: 100 μm (C). (D‐E) 293T cells were transfected with an appropriate amount of luciferase plasmids and treated with TNF‐α for 6 h, luciferase assay was performed to analyze NF‐κB transcriptional activity. Data are presented as mean ± S.D, n = 3, *p < 0.05, ***p < 0.001 vs TNF‐α group. (F) SMYD2 contributed to the expressions of COX2 and iNOS

Since we observed SMYD2 enhancing the expression of A20, the relation between SMYD2 and the NF‐κB signaling pathway was investigated. Primary synovial cells transfected with SMYD2‐targeting siRNA oligos or pCDH‐SMYD2 were treated with TNF‐α at the indicated time. As expected, TNF‐α induced A20 expression was enhanced by the overexpression of SMYD2 and suppressed by the silence of SMYD2 (Figure 2A). However, it seemed paradoxical that the upregulation of A20 induced by the overexpression of SMYD2 didn't impede the activation of NF‐κB, but promoted it. Conversely, lower expression of A20 mediated by the silence of SMYD2 decreased the activation of NF‐κB, instead of stimulation reinforcement (Figure 2A). Unexpectedly, activation of MAPK kinases was significantly increased in SMYD2 overexpression group but declined in the gene silence groups compared with control group (Figure 2A). In addition, we observed a significant reduction of TNF‐α‐induced p65 nuclear translocation in SMYD2‐deficient cells, this further confirmed our results (Figure 2B). Similarly, we noticed a much higher fluorescence intensity of phosphorylated p65 in the SMYD2 overexpression cells stimulated with TNF‐α, when compared with the control cells induced by TNF‐α (Figure 2C). Meanwhile, we performed luciferase assays and found that overexpression of SMYD2 activated NF‐κB transcription dramatically and silence of SMYD2 attenuated the NF‐κB luciferase activity induced by TNF‐α (Figure 2D). Those results suggest that although SMYD2 could elevate the expression of A20 through its HMTase activity, but could finally enhance the activation of the NF‐κB signaling pathway.

The SMYD family is comprised of five members, SMYD1‐5. So, we also investigated the global members’ effects on NF‐κB signaling activation. As shown in Figure 2E, all the SMYD members could raise the NF‐κB signaling activation significantly because of the existence common the SET domain. Furthermore, we revealed that the SET domain is the most important to activation of NF‐κB stimulated with TNF‐α (Figure 2E). We also observed that the overexpression of SMYD2 contributed to the upregulation of COX2 and iNOS (Figure 2F). As for TNF‐α‐induced inflammation in synovial cell, it has also been verified that SMYD2 could interplay with NF‐κB (Figure S2A), which could be one of the reasons for the hyperactivity of NF‐κB. In addition, NF‐κB is a coordinator of innate and adaptive immune responses and is involved in multiple cellular signaling of RNA sensors including RIG‐I and TLR3 to facilitate the transcription of IFN‐β. To verify whether SMYD2 could mediate the expression of IFN‐β through NF‐κB, the IFN‐β promoter Luc‐reporter was used. In Figure S2B, the overexpression of SMYD2 dramatically increased the IFN‐β transcription activity, and which could be suppressed by the inhibitor LLY‐507 targeting SMYD2 in the RIG‐I overexpressed 293T cells insulted with the RNA virus analogues 3p‐hpRNA. Collectively, all the results indicate that SMYD2 could positively regulate the activation of NF‐κB signaling

### 2.4 SMYD2 specifically interacts with TRAF2 in the NF‐κB signaling axis

**FIGURE 3:**
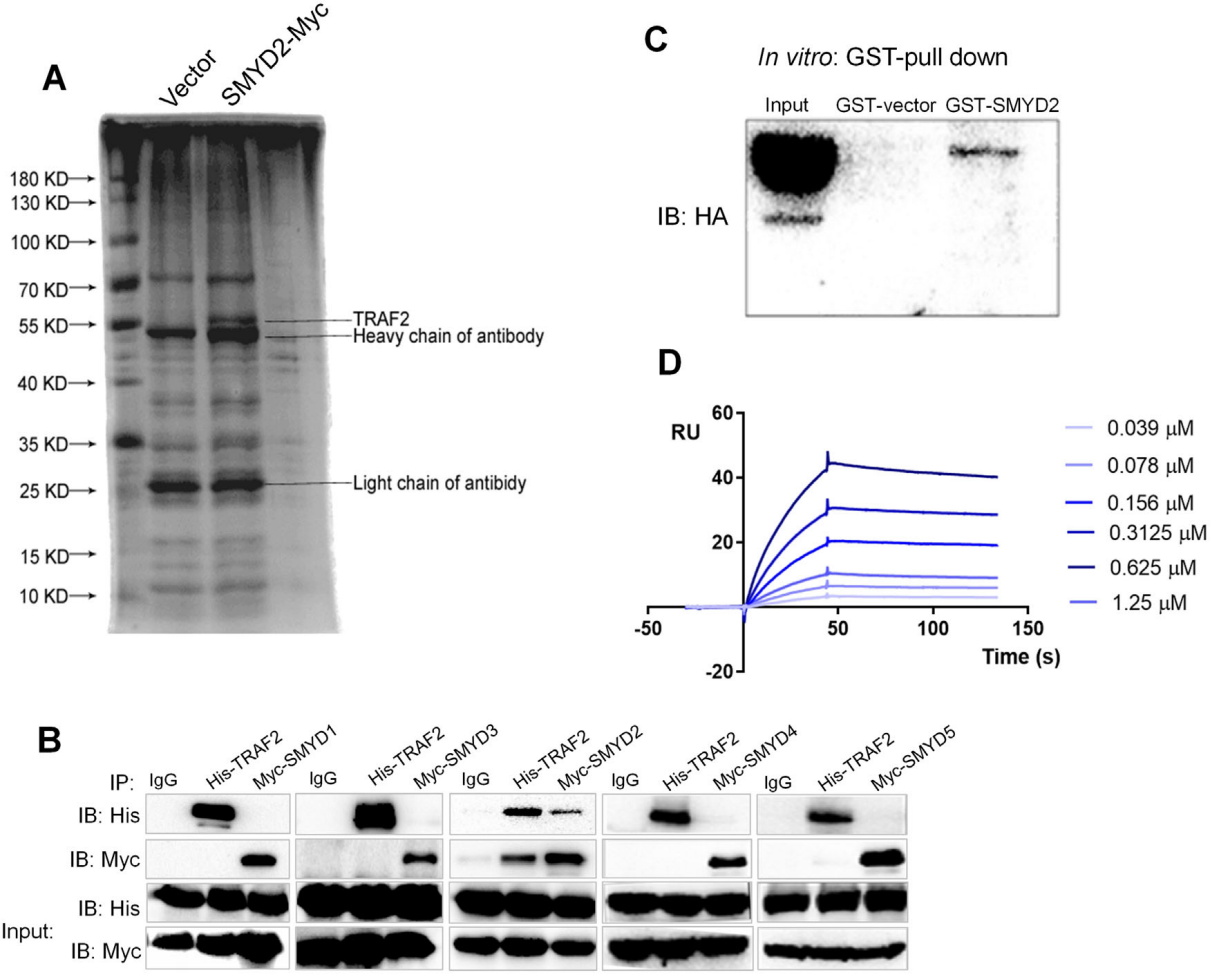
SMYD2 specifically interacts with TRAF2 in the NF‐κB signaling axis. (A) A distinct band at ∼55 kDa only in anti‐Myc‐SMYD2 samples. (B) 293T cells were transfected with His‐TRAF2, and Myc‐SMYD (1‐5) plasmids, thereafter, Co‐IP was performed using antibodies against His or Myc, respectively. (C) Western blot was performed after SMYD2‐GST pull down in vitro using full‐length recombinant HA‐TRAF2 and specific anti‐HA antibodies. Lane 1, the input corresponding to HA; Lane 2, GST alone incubated with HA; lane 3, SMYD2‐GST incubated with HA. (D) Sensorgram of the BIAcore assay showed kinetic binding curves of TRAF2 with different SMYD2 concentrations (0.039 μM ‐1.25 μM), as indicated by a different color for each concentration. RU, response units

To uncover the underlying factors involved in the NF‐κB signaling cascade that may be mediated by SMYD2, an immunoprecipitation technique combined with Mass spectrometry (MS) was used to identify interacting partners in 293T cells. We found a distinct band at ∼55 kDa only in Myc‐SMYD2 samples (Figure 3A). Each lane of the immunoprecipitated proteins derived from Myc‐SMYD2 or IgG control was excised and underwent to in‐gel tryptic digestion and identification using LC‐MS/MS. Among the identified SMYD2‐interacting proteins, we found that TRAF2 protein with a molecular mass of 55.859 kDa involved in the NF‐κB signaling cascade to form the scaffold for the binding of BIRC2 and RIPK1. In addition, we found that for all the SMYD members, only SMYD2 could interact with TRAF2 (Figure 3B). The GST‐tagged construct of SMYD2 used as bait could pull down full‐length recombinant HA‐TRAF2 in vitro (Figure 3C). Furtherly, we used Biacore assay to determine the kinetics of interaction between SMYD2 and TRAF2. Highly purified His‐SMYD2 was diluted with HBS buffer at various concentrations (0.039 μM ‐1.25 μM) and injected over His‐TRAF2‐immobilized CM5 sensor surfaces. The association of SMYD2 and TRAF2 was traced for 45 s after injection. Then replace with buffer, and the dissociation of the complex was traced for a further 100s. The curve gave a ka (1/Ms), kd (1/s), KD (M) of 3.51 × 10^4^, 1.031 × 10^−3^ and 2.94 × 10^−8^ respectively (Figure 3D). These results indicate that TRAF2 as a juncture for the NF‐κB signaling transduction could interact with SMYD2 specifically.

### 2.5 SMYD2 methylates TRAF2 on Lys 115 and Lys 194

**FIGURE 4:**
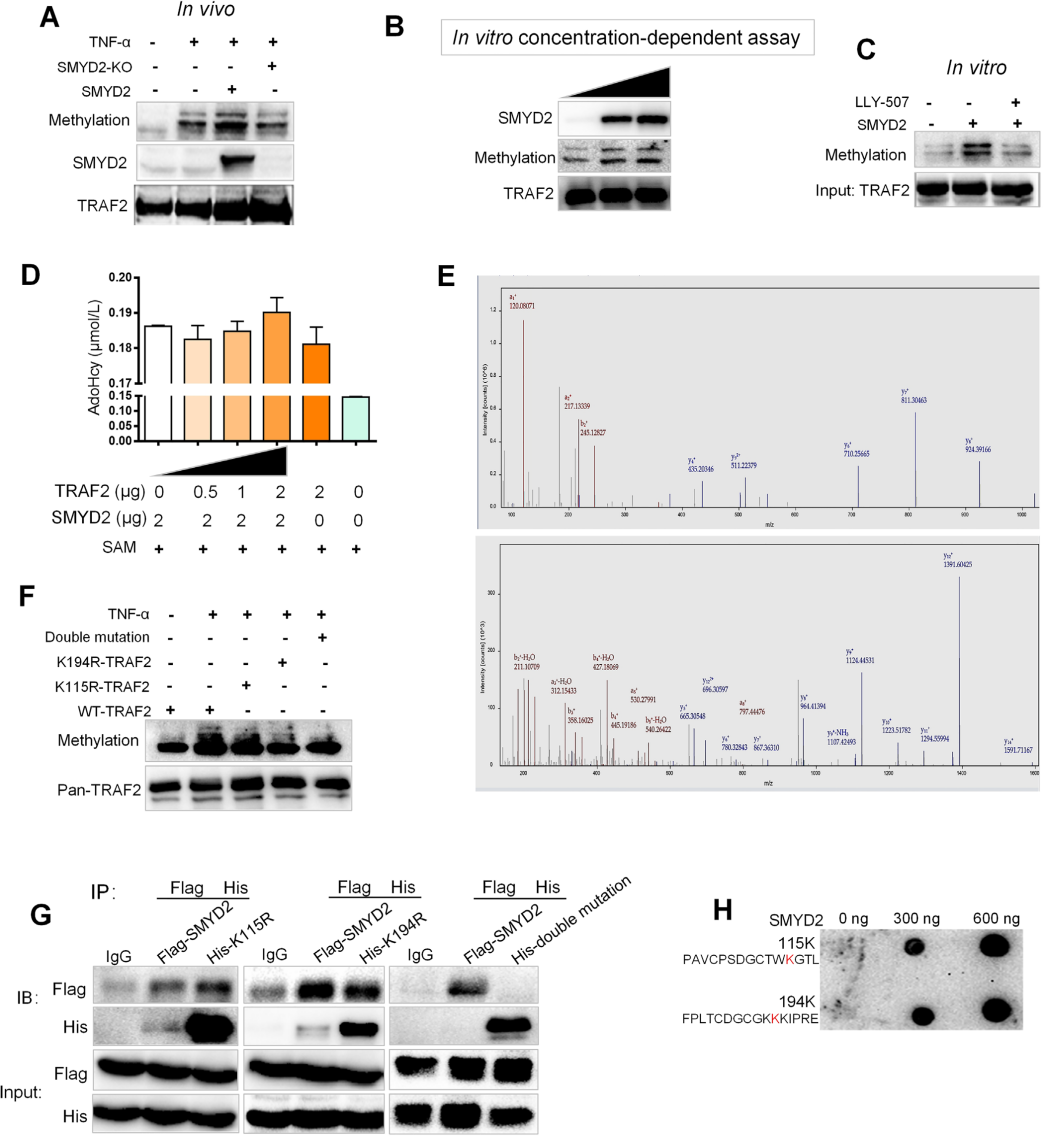
SMYD2 methylates TRAF2 on Lys 115 and Lys 194. (A) SMYD2 methylated TRAF2. SMYD2 knockout 293T cells were transfected with TRAF2, either with or without SMYD2 and stimulated with TNF‐α, TRAF2 immunoprecipitate was subjected to western blot analysis with the methylation‐specific antibody. (B) In vitro methyltransferase assays. Recombinant TRAF2 protein was incubated with the concentration gradient of recombinant SMYD2 and S‐Adenosyl methionine (SAM) in a mixture of methylase buffer for 1 h at 30°C. After denaturing, samples were separated by SDS‐PAGE. (C) LLY‐507 reduced the methylation level of the substrate TRAF2. (D) The methylation degree induced by SMYD2 was elevated with increased substrate TRAF2 concentrations. After the methylation reaction using recombinant substrate TRAF2 and SMYD2, the concentration of AdoHcy generated by SAHN and SRHH was detected. (E) Identification of monomethylated lysine sites in TRAF2 at the K115 and K194 respectively by mass spectrometry. (F) TNF‐α stimulated the methylation of TRAF2 point mutants or two mutations at the K115 and K194. (G) Co‐immunoprecipitation (Co‐IP) assay of SMYD2 and the double sites mutated TRAF2 with indicated antibodies. (H) The dot‐blot assay showed that both 115K and 194K could be methylated by SMYD2 in vitro in concentration‐dependent manner

Since SMYD2 could interplay with TRAF2, considering its methytransferase function, we next asked whether SMYD2 could methylate TRAF2. The SMYD2 knockout 293T cells were transfected with TRAF2, and stimulated with TNF‐α. The extent of lysine methylation of TRAF2 was greatly increased by cotransfection with SMYD2 and was significantly declined in SMYD2 knockout cells (Figure 4A). In vitro methyltransferase assays revealed that SMYD2 could methylate TRAF2 with the concentration‐dependent manner (Figure 4B). The 10 μM LLY‐507 as the specific inhibitor of SMYD2 could distinctly reduce the methylation level of the substrate TRAF2 (Figure 4C). S‐adenosyl‐L‐homocysteine (AdoHcy), a common product of methyltransferase, can be catalyzed into homocysteine at the presence of S‐adenosine homocysteine ribosidase (SAHN) and S‐ribosylhomocysteinase (SRHH) and finally form 5‐thio‐2‐nitrobenzoic acid (TNB) with Ellman's regents. The level of TNB shown as absorbance value represents the extent of methylation. The standard curve indicated that there was a significant linear positive correlation between TNB and initial AdoHcy concentration (Figure S3). According to the methodology mentioned above, the methylation reaction using recombinant substrate TRAF2 and SMYD2 demonstrated that the methylation degree induced by SMYD2 was elevated with the substrate TRAF2 concentration (Figure 4D). To further determine the modified sites, we subjected enriched TRAF2 proteins to mass spectrometry analysis. Eventually, we identified a total of two monomethylated lysine sits, the K115 and K194 respectively (Figure 4E). Then, we mutated the K115 and K194 to R115 and R194 and assessed the two mutations methylation induced by SMYD2 in vivo. As Figure 4F showed, the methylation degrees in two single point mutations, together with the double sites mutation, were decreased compared with the WT TRAF2, when they were stimulated with TNF‐α. Meanwhile, the interaction between SMYD2 and the double sites mutated TRAF2 was dramatically attenuated compared with the two single point mutations, which indicated that both the two lysine sites were crucial for TRAF2 methylation (Figure 4G). Furthermore, we conducted the dot‐blot assay with incubating the two segments of peptides belonging to TRAF2 and purified SMYD2 in methylation assay buffer. The results showed that both 115K and 194K could be methylated by SMYD2 in vitro in concentration‐dependent manner (Figure 4H). Those findings confirm that SMYD2 could methylate TRAF2 on Lysine 115 and Lysine 194.

### 2.6 SMYD2 induced TRAF2 methylation mediates the K63 ubiquitin of RIPK1

Owning to the function of TRAF2 attaching K63‐linked polyubiquitin chains to RIPK1 in NF‐κB signaling transduction, here we evaluated the effects of TRAF2 methylation induced by SMYD2 on RIPK1. RIPK1 represents the critical molecule working as the signaling platform by accepting K63‐linked polyubiquitin chains to activate the IKK complex upon TNF invasion. After TNF binding to TNFR1, TRADD provides a scaffold for the assembly of TRAF2, BIRC2/3 and RIPK1, then TRAF2 associated with BIRC2 acts as E3 for K63‐linked ubiquitination for NF‐κB activation. Notably, TNF stimulation caused the ubiquitination of RIPK1 within 15 min (Figure S4A). To explore which polyUb chain was influenced by SMYD2, we examined both the K48 and K63 chains attached to RIPK1 through transfecting 293T cells with pCMV‐HA‐K48 and pCMV‐HA‐K63 plasmids respectively. We observed that overexpression of SMYD2 could significantly elevate the K63 chains modification on RIPK1 compared with the vehicle group stimulated with TNF‐α (Figure S4B). However, overexpression of SMYD2 had no effects on the K48‐linked chains modification on RIPK1 (Figure S4B). When we reduced the expression of SMYD2 using siRNA targeting *SMYD2*, the K63 modification on RIPK1 was decreased accordingly (Figure S4C). To further demonstrate the effects of SMYD2 on RIPK1, the Crispr‐Cas9 system was applied in 293T cells to knockout the gene expression of *SMYD2*. We observed that loss of SMYD2 could significantly decline the level of K63 chains on RIPK1 (Figure S4D). To address whether SMYD2 effecting the ubiquitin modification on RIPK1 is K63‐linked chains specific, we used either K63 ubiquitin or K63R mutant in which internal lysine in the ubiquitin sequence had been replaced with arginine. As expected, we observed the distinct difference in RIPK1 ubiquitination between cells transfected with the K63 ubiquitin and cells transfected with K63R ubiquitin (Figure S4E). This result indicates that SMYD2 could specifically affect the K63 ubiquitin modification on RIPK1. Considering SMYD2 interacts with TRAF2, so we tried to figure out how methylation affects the function of TRAF2. The results depicted that SMYD2 could deepen the K63 ubiquitination of TRAF2 compared with SMYD2 knockout group, but decrease its K48 ubiquitin modification. (Figure S4F). Those data indicate that SMYD2 interaction with TRAF2 could strengthen the activity of TRAF2 through increasing K63 ubiquitination modification and prevent it from being degraded. Next, to further testify that SMYD2 modulates the function of TRAF2, we assessed the ubiquitin modification using mutant TRAF2. Consistent in the previous results, K63 ubiquitination level was declined in mutant group and the mutant TRAF2 could be susceptible to K48 modification (Figure S4G). As we mentioned above SMYD2 could methylate TRAF2 and proceed the K63 modification of RIPK1, next we sought to speculate whether the effects of SMYD2 on RIPK1 is dependent on TRAF2 methylation. We transfected the TRAF2 knockout 293T cells with WT or double mutant TRAF2 expression plasmids to evaluate the level of K63 linked ubiquitin modification on RIPK1. We found that there was a discernible difference between the cells transfected with WT TRAF2 and cells transfected with mutant TRAF2 (Figure S4H), which suggested that the methylation on TRAF2 induced by SMYD2 was very crucial for the K63 modification on RIPK1 when the cells were invaded by TNF‐α. Collectively, SMYD2 could reinforce the K63 ubiquitination on RIPK1 through methylation of TRAF2.

### 2.7 Methylated TRAF2 continuously activates the NF‐κB signaling axis by sustaining their own stability

**FIGURE 5:**
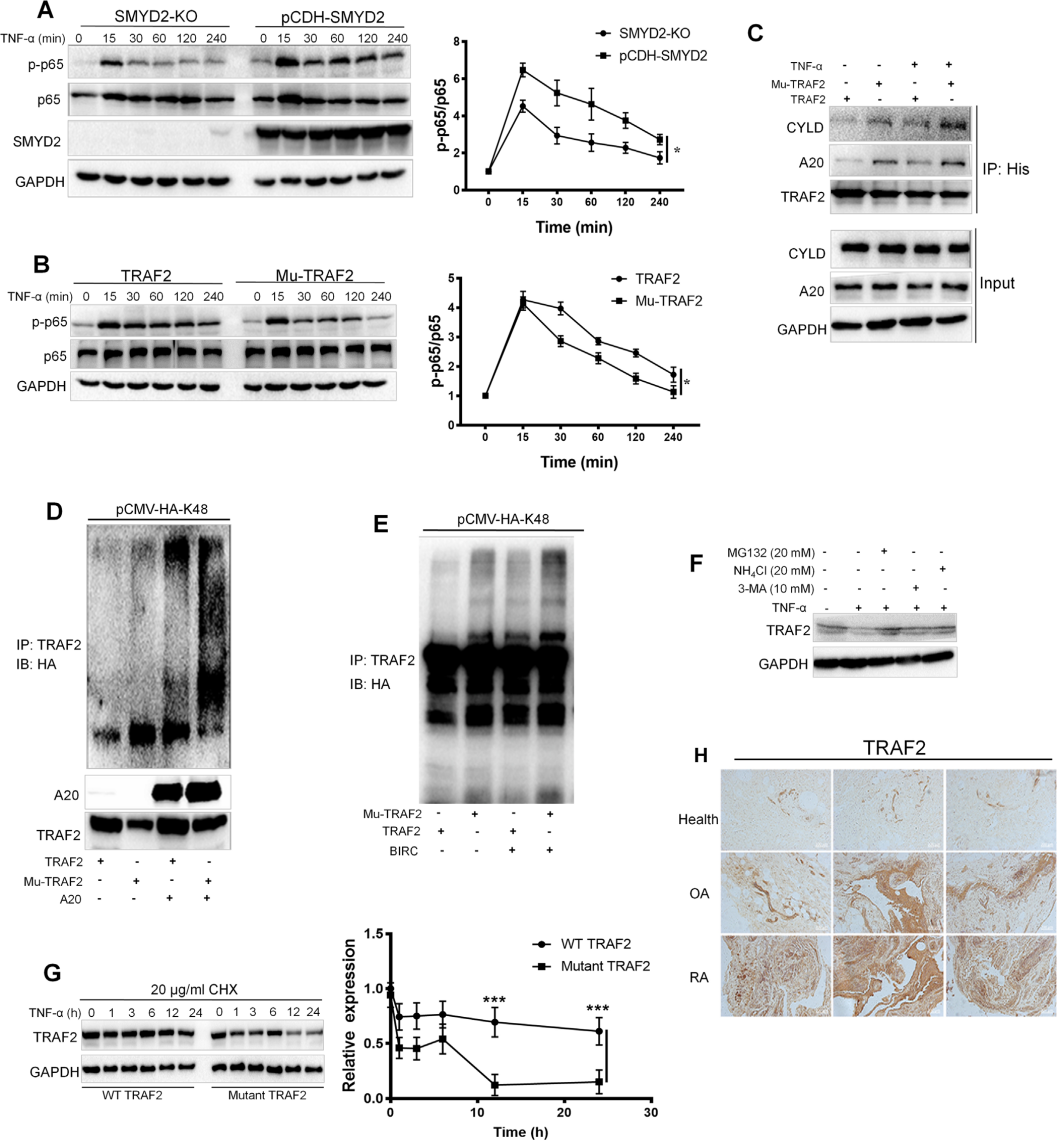
Methylated TRAF2 continuously activates the NF‐κB signaling axis by sustaining their own stability. (A) Knockout of SMYD2 impeded the phosphorylation of p65. (B) The mutant TRAF2 impeded the phosphorylation of p65. (C) The mutant TRAF2 enhanced deubiquitinating enzymes A20 and CYLD expression. Overexpressed 293T cells transfected with His‐tagged WT and the mutant TRAF2 were stimulated with TNF‐α (20 ng/ml). Cell extracts were prepared and anti‐His immunoprecipitate was analyzed by immunoblotting with anti‐CYLD and A20 antibody. (D) A20 catalyzed the K48‐linked ubiquitylation of mutant TRAF2 for degradation. (E) The mutant TRAF2 was fairly easier to be impeded by BIRC2 through K48 modification. (F) MG132 reduced TRAF2 degrading. Synovium cells were pretreated with indicated concentration MG132, 3‐MA, or NH_4_Cl, respectively, and the stimulated with TNF‐α for 6 h, cell extracts were analyzed by immunoblotting with the anti‐TRAF2 antibody. (G) Cycloheximide Chase Assay (CHX) assay showed that methylated TRAF2 has the longer half‐life. (H) Macroscopic inspection revealed a larger amount of TRAF2 in the rheumatoid arthritis group compared with the healthy group. Data are presented as mean ± S.D, n = 3, *p < 0.05, ***p < 0.001

Ubiquitination of the kinase RIPK1 is crucial in determining cell fate. The K63 ubiquitin chains attached to RIPK1 has been proofed to recruit the TAK1/TAB2/3 complex, which finally activates the transcriptional function of NF‐κB. As SMYD2 could dramatically increase the K63 modification on RIPK1, we further analyzed the phosphorylation level of p65 in SMYD2 overexpressed and SMYD2‐KO 293T cells. The intensity of activated p65 in SMYD2‐KO group was much lower than the SMYD2 overexpressed group after TNF‐α invading for 15 min (Figure 5A). Next, we also analyzed the phosphorylated p65 in WT TRAF2 and mutate TRAF2 transfected 293T cells. Consistent with the previous findings, the activation of p65 in WT group was much more strong than the mutated group, which substantiated that SMYD2 enhancing the phosphorylation of NF‐κB was dependent on the methylation of TRAF2 (Figure 5B). E3 ubiquitin ligases induced ubiquitination could be counteracted by several deubiquitinating enzymes (DUBs). For NF‐κB signaling axis, A20 and CYLD are implicated as the most important DUBs as they can cleave the K63‐specific ubiquitin chains on several kinases to restrict their activity.^18^, ^19^ Figure 5C revealed that the mutant TRAF2 had the enhanced infinity between the DUBs compared with the WT TRAF2, especially when the cells were stimulated TNF‐α. Concretely, A20 could catalyze the K48‐linked ubiquitylation of mutant TRAF2 much more easily for further degradation (Figure 5D). The results suggest that the methylated TRAF2 by SMYD2 earns stronger stability to activate the downstream factors. Besides, there were reports that BIRC2 could modulate the activity of TRAF2 by adding K48 ubiquitin chain, typically in the non‐canonical NF‐κB signaling pathway.^22^ So, we also evaluated the inhibited effects of BIRC2 on methylated TRAF2 using in vitro ubiquitination assay. Results indicated that the mutant TRAF2 was fairly easier to be impeded by BIRC2 through K48 modification (Figure 5E). Furthermore, TRAF2 together with TRAF3, BIRC2 and BIRC3 could mediate NF‐κB signaling pathway,^23^ our results showed that, compared with TRAF3, BIRC2 is much more easy to bind TRAF2 for further degradation (Figure S5). In other words, the methylated TRAF2 shows more stable resisting the degradation induced by BIRC2.

There are three major pathways identified for protein degradation, the lysosome, proteasome and autophagy pathways respectively. To address which way is preferred for TRAF2 degradation in the present of TNF‐α on synovium cells, we applied three classic inhibitors targeting different degradation. As shown in Figure 5F, MG132 could dramatically reversed TRAF2 degrading caused by TNF‐α. The results indicated that the ubiquitin‐proteasome pathway is the predominant way for TRAF2 degradation. To specifically determine the stability of methylated TRAF2, Cycloheximide Chase Assay (CHX) was used. The CHX assay showed that methylated TRAF2 has a longer half‐life compared with the mutant TRAF2 upon the stimulation of TNF‐α (Figure 5G). To consolidate the conclusion from the in vitro assay, we performed the immunohistochemistry targeting TRAF2 in synovium tissues collected from different populations. Macroscopic inspection revealed a larger amount of TRAF2 in the RA group compared with the healthy group (Figure 5H). The results suggest that the higher expression of TRAF2 indicated more malignant inflammation, the methylated TRAF2 could continuously activate the signaling pathway by keeping itself from ubiquitin induced proteasome.

### 2.8 SMYD2 maintains mature B cells survival through the non‐canonical NF‐κB pathway

Next, we determined whether methylated TRAF2 mediated by SMYD2 could regulate the non‐canonical NF‐κB pathway. The TNF‐α typically induced the presence of p52. Compared with the WT group, SMYD2 could enhance the processing of p52, on the contrary, losing SMYD2 could alleviate the activation of p52 (Figure S6A). Furthermore, we found the mutant TRAF2 could activate the non‐canonical NF‐κB pathway much faster and more severely compared with the WT group (Figure S6B). It was consistent with the report that TRAF2 could negatively regulate the formation of p52 by promoting the degradation of NIK,^24^ as the mutant TRAF2 could be eliminated by the ubiquitin system. The pieces of literature have stated that the BAFF/Blys receptor 3 could promote the survival of B cells through the processing of NF‐κB,^23^, ^24^ and which prompts us to confirm the role of SMYD2 on B cell's survival in the spleen. The Figure S6C revealed that in the *Smyd2* ^+/−^ mice, the number of mature B cells (CD19^+^ IgD^+^ IgM^+^) was less than the WT mice, but the immature B cells (CD19^+^ IgM^+^ IgD^−^) showed no difference in both groups. Next, we purified the pan B cells from mice spleen and evaluated the survival capability without any stimulation. As Figure S6D showed, loss of the expression of SMYD2 could dramatically decrease the viability of B cells. Considering B cells are the primary generator of antibodies in the body, so we tested total IgG and IgM in the serum of mice subjected to dextran sulphate sodium (DSS) induced colitis. The concentrations of both the IgM and IgG were robustly lower in the *Smyd2* ^+/−^ mice compared with WT mice (Figure S6E). To substantiate the role of SMYD2 on B cells’ survival, the pan B cells were collected from the spleens and incubated with 50 ng/ml BAFF the ligand for BAFF/Blys receptor 3. After stimulation overnight, the mRNA level of related survival genes was detected by qPCR and the results explained that most of the anti‐apoptosis genes such as *Bcl‐2*, *Xaip* and *Baff* were reduced in the *Smyd2^+/−^* mice (Figure S6F). Hence, SMDY2 could maintain mature B cells survival through the non‐canonical NF‐κB pathway and which could also explain why B cells are excessive activation in some autoimmune diseases and chronic lymphocytic leukemia accompanied by the high expression of SMYD2.

### 2.9 LSD1 as the demethylase could alleviate activation of NF‐kB mediated by SMYD2

**FIGURE 6:**
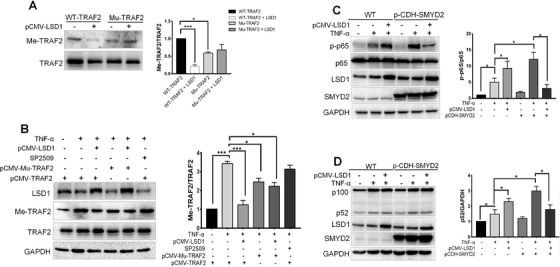
Demethylase LSD1 alleviates activation of NF‐κB enhanced by SMYD2. (A) Overexpressed LSD1 reduced the methylation level of TRAF2. WT and the mutant TRAF2 overexpressed 293T cells were transfected with LSD1 plasmids, immunoblot analyzed for Me‐TRAF2 and TRAF2. (B) LSD1 inhibitor SP2509 had not effect on TRAF2 methylation. WT and the mutant TRAF2 overexpressed 293T cells were transfected with LSD1 plasmids, and were treated with SP2509, subsequently stimulated with TNF‐α (20 ng/ml) for 24 h, immunoblot analyzed for LSD1, Me‐TRAF2, and TRAF2. (C‐D) Overexpression LSD1 downregulated activation of the NF‐κB pathway in SMYD2 overexpressed cells. WT and SMYD2 overexpressed 293T cells were transfected with LSD1 plasmids and stimulated with TNF‐α (20 ng/ml) for 15 min or 12 h, immunoblot analyzed for LSD1, p‐p65, p65 and SMYD2 (C); Immunoblot analyzed for LSD1, p52, p100 and SMYD2 (D). All data are presented as mean ± S.D, n = 3, *p < 0.05, ***p < 0.001

Lysine‐specific demethylase 1 (LSD1) is the first enzyme identified to remove the methyl group from methylated lysines on histone proteins.^27^ We investigated whether LSD1 could demethylate TRAF2 in the NF‐κB signaling axis. Overexpressed LSD1 in 293T cells could significantly reduce the methylation level of WT TRAF2 but showed no effects on mutant TRAF2 (Figure 6A), which suggested that LSD1 could actually demethylate TRAF2 on the same sites that SMYD2 targeted. To further confirm the results, we used LSD1 specific inhibitor SP2509 to treat the 293T cells, but SP2509 seemed to have no effect on TRAF2 methylation (Figure 6B). Further, we found the aberrant expression of LSD1 or SMYD2 could enhance the activation of the canonical and non‐canonical pathway. However, the activation of the NF‐κB pathway was dramatically downregulated, when SMYD2 and LSD1 were both overexpressed (Figure 6C and D). We postulated that only in the condition of high expression of SMYD2 induced by TNFR, LSD1 could attenuate the NF‐κB pathway activation.

### 2.10 SMYD2 knockdown and SMYD2 inhibitor hinder the inflammation process

**FIGURE 7:**
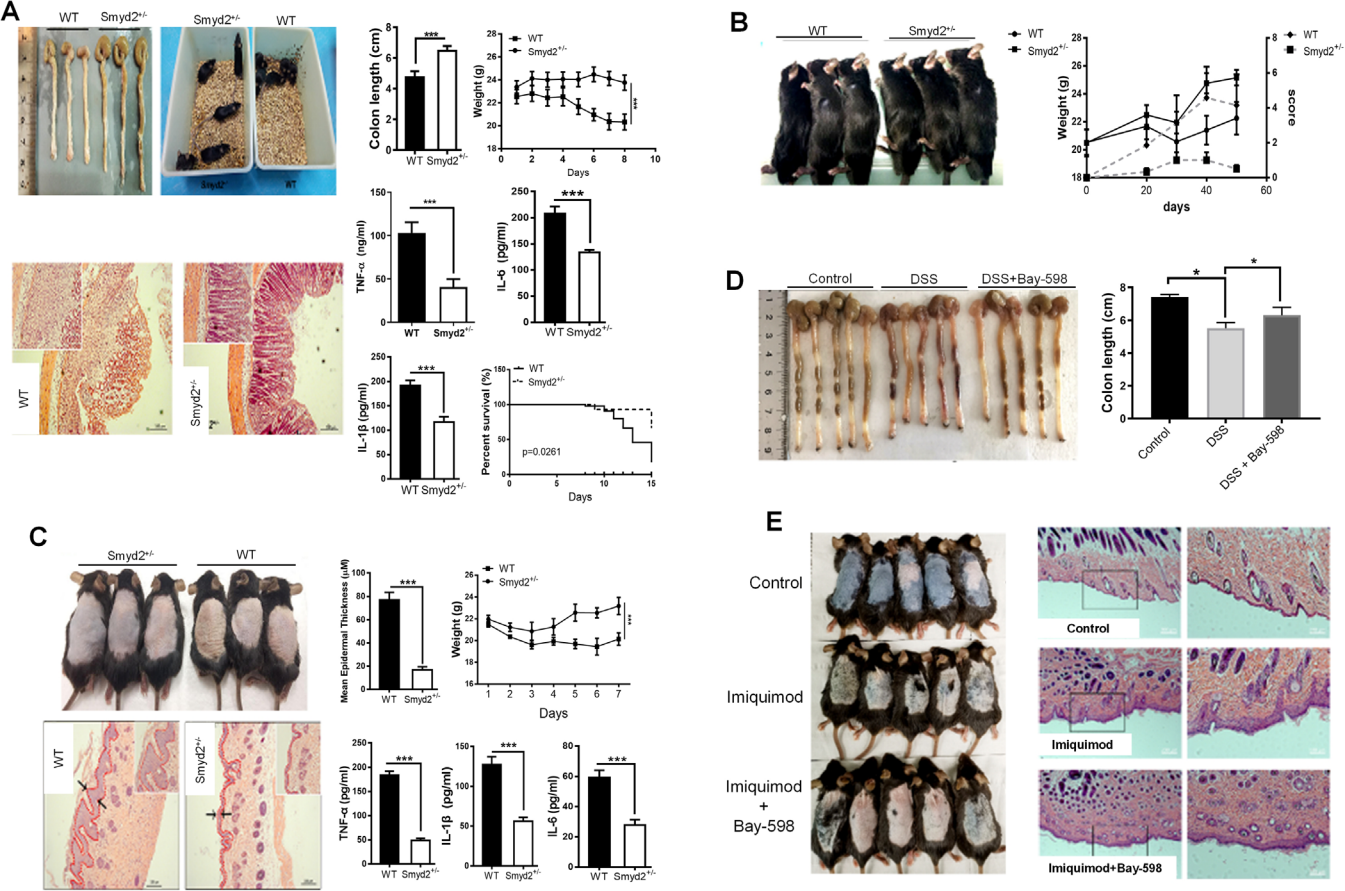
SMYD2 knockdown mice and SMYD2 inhibitor hinder the inflammation process. (A) *Smyd2* ^+/‐^ mice showed robust tolerance on the DSS model. (B) *Smyd2* ^+/‐^ mice were much more retarded to Body weights in collagen‐induced arthritis (CIA). (C) S*myd2* ^+/‐^ mice showed powerful resistance to psoriasis. (D) SMYD2 selective inhibitor Bay‐598 reduced the inflammation process in DSS‐induced colitis mice model. (E) H&E staining of back skin showed Bay‐598 reduced the inflammation process in psoriasis mice model. All data are presented as mean ± S.D, n = 5, *p < 0.05, ***p < 0.001

Next, we sought to determine the impact of SMYD2 on acute and chronic inflammation within different mice models. We firstly used the *Smyd2* ^+/‐^ mice to evaluate the roles of SMYD2 on colitis, collagen‐induced arthritis (CIA) and imiquimod induced psoriasis. Initially, western blot determined that SMYD2 levels remained low in main organs and macrophages of *Smyd2* ^+/‐^ mice compared to the WT mice (Figure S7). The colon length in *Smyd2* ^+/−^ group was significantly longer than in the colitis WT mice. Besides, the body weights of *Smyd2* ^+/−^ mice were much higher and the occult blood symptom is more mitigatory compared with the WT group. H&E staining displayed the DSS led to colonic ulceration associated with the inflammatory cell infiltration and large areas of epithelial crypt loss in the WT mice. Meanwhile, *Smyd2* ^+/‐^ mice had powerful resistance to the DSS induced inflammation with the longer survival period (Figure 7A). We used RA mouse as the chronic inflammation model. The *Smyd2* ^+/‐^ mice were much more retarded to collagen‐induced arthritis, however, the WT mice lost more body weights and exhibited markedly exacerbated inflammation effects (paw inflamed and swollen) (Figure 7B). Furtherly, we conducted the imiquimod‐induced psoriasis mice model. As the Histological assessment of back skin by H&E staining and the ELISA assay demonstrated that the *Smyd2* ^+/‐^ mice showed powerful resistance to psoriasis (Figure 7C). It has been testified that the Bay‐598 is a highly potent and selective inhibitor for SMYD2, the treating with 50 mg/kg Bay‐598 could substantially reduce the inflammation process in DSS‐induced colitis mice model and the psoriasis mice model (Figure 7D and E).

The dysfunction of B cells plays pivotal roles in autoimmune diseases. As we uncovered that SMYD2 could prolong the survival of matured B cells through the noncanonical NF‐κB signaling pathway. So, we reasoned that the excessive activation of B cell caused by the anomalous expression of SMYD2 could contribute to the continuous inflammation. We analyzed the *Smyd2* ^fl/fl^ and *Smyd2* ^fl/fl^ *CD19*‐Cre mice with acute colitis induced by DSS. As shown in Figure S8A and B, the colon lengths in *Smyd2* ^fl/fl^ *CD19*‐Cre mice were pronouncedly longer. H&E staining demonstrated that the administration of DSS leaded to colonic ulceration associated with a larger range of epithelial crypt loss and more inflammatory cell infiltration into the mucosa in the *Smyd2* ^fl/fl^ mice. We observed a marked decrease in the expression of CD19 in the spleen from *Smyd2* ^fl/fl^ *CD19*‐Cre mice, further substantiating the finding (Figure S8C). All those above verify that anomalous expression of SMYD2 enhances the inflammation by sustaining and overactivation of B cells.

## 3 DISCUSSION

NF‐κB signaling pathway has been uncovered to have a broad role in gene activation or repression in numerous biological processes. In most resting cells, NF‐κB is inactive and arrested in the cytoplasm by IκB protein. For the canonical NF‐κB pathway activation, it depends on the degradation of IκBs, especially IκBα and which is mediated by IκB kinase (IKK) induced phosphorylation.^26^, ^27^ The noncanonical NF‐κB pathway activation relies on the distinct mechanism that needs p100 processing to p52, which is regulated by polyubiquitination and proteasomal degradation.^23^ TRAFs work as the scaffold to transmit the signaling from the membrane receptors including TNFR superfamily and the interleukin‐1 receptors to downstream kinases activation. TNFR1 contains the intracellular death domain that could recruit the adapter protein TRADD and TRADD and further assemble TRAF2 to form the platform for the binding of RIPK1. For TNFR2, which lacks the intracellular death domains, could recruit the TRAF directly through short sequences in their intracellular tails.^30^ The N‐terminal portion of the TRAF family contains a RING finger and several zinc finger motifs and all which is crucial for the downstream signaling cascade. The RING domain is wildly believed to be essential for RIPK1 polyubiquitination.^31^ In our results, we also determined that the overexpression of TRAF2 could significantly upregulate the K63 ubiquitination of RIPK1 followed by the activation of IKK, when TNFR1 was activated by TNF‐α. As for the TNFR2 signaling pathway, it is reported that TRAF2 activates BIRC2/3 by the K63 ubiquitination to initiate the degradation of TRAF3 so as to facilitate NIK stabilization and NF‐κB‐p100 processing.^31^, ^32^ As the junction for the canonical and non‐canonical NF‐κB signaling cascade, the accurate mediation of the function of TRAF2 in cells is elaborate.

SMYD2 belongs to the Smyd family and is known for its methylation of H3 at lysine 4 or lysine 36 as the methyltransferase with the results of specific genes transcription. Recently, much more information about its methylation of the nonhistone proteins was uncovered and which implies multiple functions of SMYD2 in physical or pathological conditions.^7^, ^33^ SMYD2 could directly interact with NF‐κB and enhance its transcriptional activity by methylation of K310 on NF‐κB in the autosomal dominant polycystic kidney disease.^6^ In our study, we found that a higher level of SMYD2 exists in the synovium of RA patients and in the synovial cells insulted with TNF‐α. What's more, we also uncovered that overexpression of SMYD2 could elevate the phosphorylation of IκB, an upstream kinase of NF‐κB, which indicated that SMYD2 might interact with some other proteins located to the NF‐κB signaling pathway. Further research suggested the E3 ubiquitin‐protein ligase TRAF2 could be methylated by SMYD2 on K115 and K194. Many pieces of evidence show TRAF2 is a necessary factor for the activation of NF‐κB triggered by TNF‐α, and TRAF2 could directly polyubiquitinate RIPK1, leading to NF‐κB activation. But more and more kinds of literature demonstrate that the procedure is much more complicated and needs other cofactors such as BIRC2/3. Li *et al* showed that the phosphorylation of TRAF2 by PKC mediated its K63‐linked polyubiquitination.^36^ Collectively, the K63‐linked polyubiquitination of TRAF2 is a key mediator in TNF signaling. Unprecedently, we uncovered that SMYD2‐induced methylation of TRAF2 could boost the phosphorylation of NF‐κB due to the augmented stability of methylated TRAF2, preventing it from proteolysis. Considering some other deubiquitinases such as USP48 and UCHL3 have the ability of removing K48‐ligase from TRAF2 in pathological condition,^35^, ^36^ we also evaluated the transcriptional level of these two factors and found that overexpression of SMYD2 could hamper their transcription (Figure S9). The controversial results might be reasoned that SMYD2 induced methylation on TRAF2 alters its configuration and changes the affinity between TRAF2 and other proteins, which further unravel the significant influence of methylation on TRAF2 induced by SMYD2. As the main deubiquitination of various molecules in the NF‐κB pathway, CYLD and A20 negatively regulate NF‐κB transcription factor‐mediated gene expression. Our study exhibited high expression of SMYD2 in synovial cell stimulated by TNF‐α could actually increase the transcription of A20 by increasing H3K4me on the promoter region of A20, but instead of the suppression of the activation of NF‐κB, which reminded us that the effect of methylated TRAF2 modulated the NF‐κB cascade predominantly. As A20 expression is related to NF‐κB activation, we concluded that the overexpression of A20 induced by SMYD2 was a negative feedback withstanding the irritation of NF‐κB. IP assay results indicated that the methylated TRAF2 weakened the interaction with A20 or CYLD. It has also been proved that BIRC2 is responsible for the K48‐ubiquitination of TRAF2 after TNF‐α insult. Here, we showed that the mutant TRAF2 on K115R and K194R was susceptible to the K48‐ubiquitination by BIRC2. Collectively, those data affirm that the methylated TRAF2 by SMYD2 could exactly improve its own stability so as to continuously activate NF‐κB pathway with prolonged phosphorylation level of NF‐κB.

As the hinge point of the canonical and noncanonical signaling pathway, TRAF2 together with TRAF3, BIRC2 and BIRC3 could suppress the noncanonical NF‐κB signaling pathway in resting cells by targeting NIK for degradation.^37^, ^38^ It has been reported that CD40 or BAFF induced activation is dependent on the Lys63‐linked polyubiquitination of TRAF2, and TRAF2‐KO spleen B cells from TRAF2 deficiency mice showed higher activation of p100 processing to p52.^39^ Here, we found that the K115R and K194R mutant TRAF2 could also enhance the noncanonical NF‐κB pathway compared with the wild type TRAF2, which could be due to mutant TRAF2 damaged the accumulation of NIK as the core module in this assembly, resulting in the easier degradation of the mutant TRAF2 by proteolysis. However, a point should be mentioned that the overexpression of SMYD2 could also increase the accumulation of p52. We speculated that SMYD2 could strengthen the E3 ligase activity of TRAF2, so as to promote BIRC2/3‐TRAF3 cascade, releasing the NIK from TRAF3. Meanwhile, it could not be ruled out that SMYD2 might interact with another protein belonged to NF‐κB signaling pathway, such as NIK or p52 directly. The maturation of B cells is highly dependent on the alternative NF‐κB pathway. So, we also evaluated the effects of SMYD2 on B cells and got the conclusion that *Smyd2* heterozygous mice owed much less matured B cells in the spleen, and the survival of B cells was also shorter compared with the wild type mice. In a word, the modulation effects of TRAF2 on NF‐κB activations are much more complicated and elaborate.

As the first identified histone demethylase LSD1 has proved to demethylate the non‐histone proteins methylated by SMYD2. For example, the tumor suppressor p53 could interact with LSD1 and be demethylated on K370me induced by SMYD2.^40^ So, we explored whether LSD1 could resist non‐histone protein TRAF2 methylated by SMYD2. Our results indicated that LSD1 could specifically remove the methylation on TRAF2 induced by SMYD2 and so as to relieve the activations of NF‐κB in the inflammation model. In other reports, LSD1 was testified as the accelerator of inflammatory response.^41^ However, in our research, we uncovered that in the condition of high expression of SMYD2, the evoked inflammation responses could be suppressed by LSD1, sole overexpression of LSD1 actually promotes the inflammation responses. The paradoxical results might arise from the multiple demethylation effect of LSD1 on NF‐κB signaling pathway components and it seems more sensitive to demethylate substrates methylated by SMYD2, so more evidence should be presented in the future.

## 4 CONCLUSIONS

Our results show that the overexpression of SMYD2 could promote both the canonical and non‐canonical NF‐κB pathway in the inflammation diseases. And firstly, we point out that TRAF2 could interact with SMYD2 and be methylated by SMYD2, so as to promote the NF‐κB activation, and which could be antagonized by LSD1 specifically. Our data provide the novel clues for the treatment of inflammation diseases.

## 5 MATERIALS AND METHODS

Detailed methods can be found in the Supplementary materials 1.

### 5.1 Experiments with mice

All the animal operation was complied with the guiding principles of laboratory animal care formulated by Fudan University. Eight‐week‐old male C57bl/6J mice were purchased from SLAC Laboratory Animal Co.,Ltd, Shanghai China. *Smyd2* ^+/‐^ heterozygous mice and *Smyd2* ^fl/fl^ mice were obtained from Institute of Model Animal, Wuhan University. They were crossed with *Cd19*‐Cre mice to generate *Smyd2* ^fl/fl^ *Cd19*‐Cre mice. Control mice for all experiments in this study were littermate *Smyd2* ^fl/fl^ mice lacking the *Cre* transgene. Mice were housed in individually cages fed a standard diet with freely available food and water. All the mouse strains were used at an age range of 8–10 weeks.

For induction of collagen‐induced arthritis (CIA) in C57 mice, chick type II collagen (Chondrex) emulsified in an equal volume of Freund's complete adjuvant (CFA) (Chondrex). Mice were immunized with 100 μl of the emulsion intradermally at the base of the tail. At day 21 after the first immunization, mice received another booster injection with an emulsion of collagen and CFA. Arthritis will develop in 28–35 days following the first immunization. Acute colitis was induced by treatment of mice with 3.5% (w/v) DSS (MP Biomedicals) (MW 36–50 kDa) dissolved in sterile, distilled water for 7–9 days. The mice received the inhibitor as indicated by oral administration. For the mouse model of psoriasis, mice were administered a daily topical dose of 62.5 mg of the cream containing 5% imiquimod (Mingxinlidi Laboratory) on hair‐free back of mice for 7 days and treated with the inhibitor as mentioned before.

### 5.2 Statistical analysis

All data were presented as the means ± SD and all data analysis was done by GraphPad Prism 5 software. Statistical analyses were performed using one‐way analysis of variance with Tukey's test for *post hoc* comparisons and Student's t‐test was applied when comparing between two groups. ^*^ *p* < 0.05, ^**^ *p *< 0.01, ^***^ *p *< 0.001.

## AVAILABILITY OF DATA AND MATERIALS

All data in our study are available upon request.

## ETHICS APPROVAL AND CONSENT TO PARTICIPATE

The Research Ethics Committee of Fudan university granted ethical approval for the use of human subjects. All participants gave informed consent to be included in the study.

Animal studies in nude mice were approved by the Institutional Animal Experiment Committee of Fudan university and carried out in accordance with the “Guide for the Care and Use of Laboratory Animals” prepared by the National Academy of Sciences and published by the National Institutes of Health (NIH publication 86‐23 revised 1985).

## CONFLICT OF INTEREST STATEMENT

The authors of this manuscript have no conflicts of interest to disclose.

## Supporting information

Supporting information
