# Supplementary material for: SMYD2‐mediated TRAF2 methylation promotes the NF‐κB signaling pathways in inflammatory diseases
Source: Clin Transl Med. 2021 Nov 4;11(11):e591. doi: 10.1002/ctm2.591 (PMC8567046; doi:10.1002/ctm2.591)
Supplement: Supplementary file 1 — Supporting information [file CTM2-11-e591-s001.docx]

**Supplementary material**

**Materials and methods**

The following antibodies and reagents were purchased from commercial sources: The RIPK1 (3493), NF-κB2 p100/p52 (3017), NF-κB p65 (8242), Phospho-NF-κB p65 (3033), SMYD2 (9734), COX2 (12282), Mono-methyl-Histone H3 (Lys4) (5326), Lamin A/C (4777), A20/TNFAIP3 (5630), Phospho-JNK (4668), Phospho-p44/42 MAPK (Erk1/2) (4370), Phospho-IκBα (2859), iNOS (39898) antibodies were obtained from Cell Signaling Technology; methylated lysine (di methyl, mono methyl) antibody (HRP) (ab23367) was purchased from Abcam; BIRC2 (10022-1-AP), SMYD2 (21290-1-AP), TRAF2 (26846-1-AP), GAPDH (60004-1-Ig), KDM1 (20813-1-AP); DDDK tag (Flag) (66008-3-Ig), 6*His tag (66005-1-Ig), HA tag (51064-2-AP), MYC tag (60003-2-Ig), GST tag (66001-2-Ig) antibodies were obtained from Proteintech Group. Chemical reagents used in this study were as follows: dimethylsulfoxide ( Sigma-Aldrich, D2650), ammonium chloride (Sigma-Aldrich, A9434), dextran sulfate sodium (MP Biomedicals, 02160110), Cycloheximide (Cell Signaling Technology, 2112), 3-Methyladenine (MedChemExpress, HY-19312), SP2509 (MedChemExpress, HY-12635), LLY-507 (MedChemExpress, HY-19313), BAY-598 (MedChemExpress, HY-19546), Cell Counting Kit-8 (MedChemExpress, HY-K0301), MG-132 (Sigma-Aldrich, HY-13259), IPTG (MedChemExpress, HY-15921). Biological reagents applied were as follows: minimun essential medium (Gibico, 11090081), Complete Freund’s Adjuvant (Chondrex, 7009), Collagen (Chondrex, 20012), Recombinant human TNF-α (PeproTech, 300-01A), Recombinant rat TNF-α (PeproTech, 400-14), Recombinant human BAFF (PeproTech, 310-13), 3p-hpRNA (InvivoGen, tlrl-hprna).

**Cell culture**

Human embryonic kidney (HEK) 293T cells obtained from the American Type Culture Collection were maintained in Dulbecco’s modified Eagle’s medium (DMEM) (Invitrogen) supplemented with 10% fetal bovine serum (FBS) (Gibco) and antibiotics (Gibco) at 37°C under 5% CO_2_. Primary human or rat synovial fibroblast cells isolated from the synovial tissue using the collagenase digestion method were cultured in RPMI 1640 (Invitrogen) supplemented with 10% FBS and antibiotics in humidified 5% CO_2_ in air 37°C. Transfection was performed using Lipofectamine 2000 (Thermo Fisher) for plasmid or Lipofectamine-iMAX (Thermo Fisher) for siRNA according to the manufacture’s instruction. Unless indicated, cells were harvested between 36 and 48 h after transfection and testified by Western blotting and immunoprecipitation.

**Small interfering RNA (siRNA) and plasmids construction**

siRNA targeting human *Smyd2* and a negative control were purchased from GenePharma (Shanghai, China). The sequences are as follows: *Smyd2* siRNA: 5’- GAUUUGAUUCAGAGUGACA-3’ and the scrambled siRNA: 5’-AATTCTCCGAACGTGTCACGT-3’. Plasmids constructed in this study were listed in the Table S1. All the plasmids were confirmed by the DNA sequencing method.

**Construction of SMYD2 and TRAF2 knockout stable 293T cell lines**

We used CRISPR/Cas9 system to generate *SMYD2* and *TRAF2* knockout stable cell lines. The guide RNAs targeting SMYD2 or TRAF2 designed by the online tool (<http://crispr.mit.edu/>) were cloned into the px459 vector under the hU6 promoter. The vectors were expressed in 293T cell line. 48 hours after transfection, the cells were selected by puromycin. The remaining cells were detached and transferred to 48-well plates by serial dilution. After the cell forming confluence, cells were passaged and parts of the cells were subjected to DNA extraction, PCR amplification and sequencing. Cells containing the genomic alternation at the gRNA targeting sits were cloned for further study. Other plasmids constructed were listed in Table S1.

**TRAF2 or its mutant reintroduction**

Recombinant lentivirus vectors containing *TRAF2* or its mutant sequences were constructed by one-step recombinant cloning and the lentiviruses were packaged according to the standard procedure. The *TRAF2* knockout cell line was infected with the lentiviruses in the presence of 10 μg/ml polybrene (Sigma-Aldrich).

**Co-immunoprecipitation**

After the stimulation, cells were rinsed with fresh PBS and lysed in immunoprecipitation buffer [25 mM tris-HCl (pH 7.4), 5% glycerol, 150 mM Nacl, 1 mM EDTA and 0.5% NP-40] including a protease inhibitor cocktail (Roche) on ice. Lysates were cleared by centrifugation (input samples were taken), protein levels were quantified and homogenized. The antibodies indicated in the study were incubated with the lysate samples overnight with rotation at 4°C. 50 μl of a slurry of protein A/G-Sepharose beads (Santa Cruz) were added to the lysate for further 2 h. Precipitates were washed three times with the immunoprecipitation buffer and resuspended in the 1x loading buffer for SDS-PAGE analysis.

**Western blot analysis**

Whole-cell protein extracts were prepared in RIPA buffer with complete protease inhibitors and quantified using the BCA method (Thermo Fisher Scientific). The samples were boiled in 1x sodium dodecyl sulfate buffer for 7 minutes. The protein lysates were resolved via PAGE and transferred to nitrocellulose membranes (Millipore). The membranes were blocked with 5% skim milk and then incubated overnight at 4°C with specific primary antibodies. The second antibody was added in the membranes, incubated for 2 hours after Tris buffer saline-tween (TBST) washing. Finally, the enhanced chemiluminescence developing solution (Millipore) was added to membrane, and the images were recorded and analyzed.

**RNA extraction and quantitative real time PCR analysis**

Total RNA was isolated from the cells or tissues using TRIzol reagent (TAKARA). Reverse transcription was performed according to the instruction of Prime RT Master Mix kit (TAKARA). Quantitative real-time PCR was performed in triplicate using SYBR Premix EX Taq II (TAKARA). The sequences of primers used in the research were shown in Table S2.

**Immunohistochemical staining**

4-μm sections of paraffin-embedded whole tissues sections were pretreated with autoclaving at 121°C for 15 min in 0.01 mol/L citrate-buffered saline (pH 6.0) for antigen retrieval. The sections were then immersed in 3% H_2_O_2_ for 30 min at room temperature to block the endogenous peroxidase activity. After deactivation, 10% normal goat serum was used to block nonspecific binding of the immunological reagents. After incubation of the antibodies against TRAF2 and SMYD2 at 4°C overnight, each slide was rinsed three times in PBS and incubated with biotinylated anti-mouse IgG and HRP-streptavidin at room temperature according to the immunohistochemical staining kit (Beyotime Biotechnology), stained with DAB substrate. Finally, nuclear counterstaining was done using hematoxylin.

**Immunofluorescence**

After the stimulation as indicated, cells were fixed with pre-warmed 4% paraformaldehyde and then permeabilized for 5 min using 1% Triton X-100 in dissolved in PBS followed by incubation with the primary antibodies at 4°C overnight. The cells were washed three time with PBS and incubated with Fluor-conjugated secondary antibody (Invitrogen). The nucleus was staining with 4',6-diamidino-2-phenylindole (DAPI) to locate the cell. Fluorescent pictures were obtained with Zeiss fluorescence microscope.

**Mass spectrometric assay**

To identify the binding proteins interacting with SMYD2, we overexpressed the protein SMYD2 through transfecting 293T cells with plasmid PCMV-C-FLAG-SMYD2 and the PCMV-C-FLAG as the control. After the invasion of TNF-α, we immunoprecipitated the SMYD2 using anti-Flag immunomagnetic microbeads (Bimake) according to the illustration. The immunoprecipitation was separated by SDS-PAGE. Sliver staining was used to present the proteins on gel. The protein bands were detected by mass spectrometry according to the standard procedure.

To identify in vivo methylation sites of TRAF2, 293T cells were transfected with His-tagged TRAF2. After 48 hours, the cell lysate was collected. His-TRAF2 was immunoprecipitated using Ni-NTA resin and subjected to SDS-PAG. The gel was stained with coomassie blue. The band corresponding to TRAF2 was subjected to in-gel trypsin digestion. The resulting peptides were extracted from gel and separated on C_18_ column. The samples were analyzed by LC-MS/MS and the resulting data was processed with Proteome Discoverer (Thermo Fisher Scientific) and searched against the Swiss-prot Homo sapiens protein sequence database.

**In vitro methyltransferase assay and ubiquitination assay**

Recombinant TRAF2 protein was incubated with recombinant SMYD2 and methionine (New England Biolabs) in a mixture of methylase buffer (50 mM Tris-HCl pH=8.8, 10 mM DTT and 10 mM MgCl_2_) for 1 hour at 30°C. After denaturing, samples were separated by SDS-PAGE.

For the ubiquitination reaction, 5 μg of purified TRAF2 was incubated with 0.1 μg human recombinant E1 Ub-activating enzyme His-UBE1 (UB Biotechnology), 0.2 μg of purified E2 enzyme CDC34 (UB Biotechnology), 1 μg recombinant BIRC2 and 1 μg of ubiquitin (UB Biotechnology) in 25 mM Tris-HCl (pH=7.4), 100 mM NaCl, 5 mM MgCl_2_, 1 μM dithiothreitol, and 2 mM ATP at 30°C for 1 hour. The ubiquitin resulted was confirmed by SDS-PAGE.

**Luciferase reporter assays**

The human IFN-β promoter pGL3 (Basic) luciferase plasmid containing IFN-β promoter fragment was purchased from MiaoLingBio and the human NF-κB promoter luciferase plasmid was purchased from Yeasen Biotech. 293T cells were transfected using lipofectamine 2000 with the related luciferase constructs that encodes firefly luciferase reporter genes, a control vector containing Renilla luciferase (Promega). After the transfection and stimulation, luciferase activity was assessed using a dual luciferase assay kit (Beyotime Biotechnology).

**ChIP-PCR**

ChIP assays was performed as described previously. Briefly, the cells were fixed with 1% formaldehyde for 10 min, then neutralized with 0.125 mM glycine. Then, the samples were sheared with sonication. DNA was isolated after immunoprecipitation with the specific Methylated H3K4me (Cell Signaling Technology) and a rabbit IgG (Santa Cruz) antibodies. Normal PCR was applied to quantify precipitated DNA using rat A20 promoter-specific primers (Forward: 5’-GAACCTTGCCACTGAACC-3’; Reverse: 5’-TCCTCCACCTTTGATTGC-3’).

**
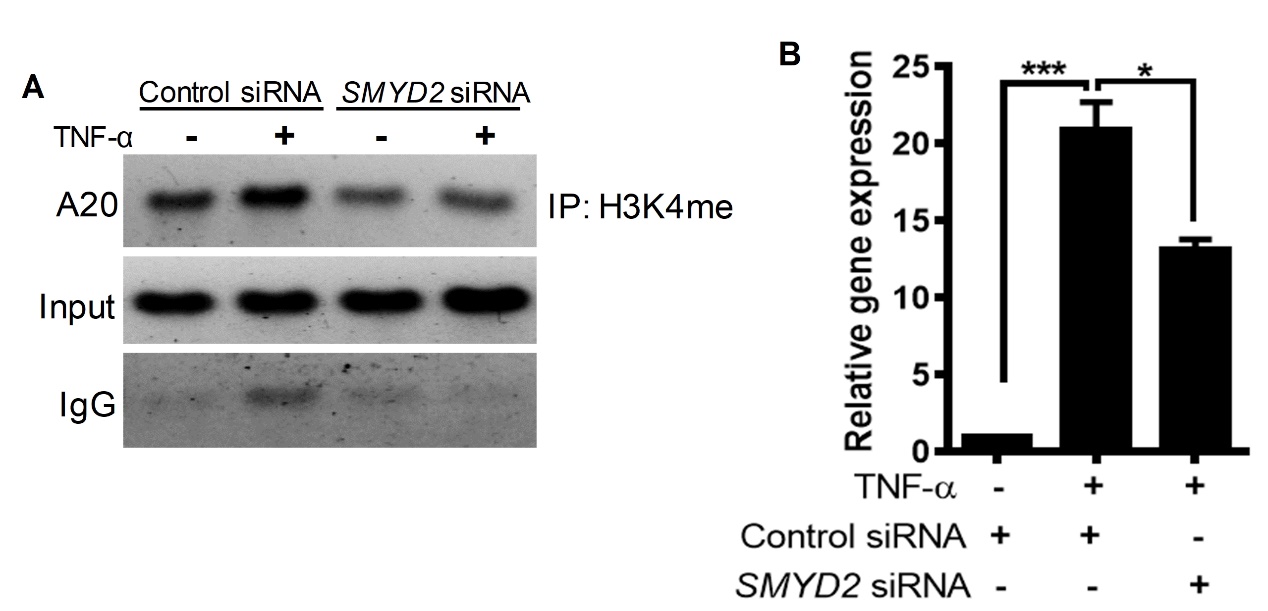
**

**Fig. S1 Binding of SMYD2 to promoters of A20 causes increased H3K4me level and elevated gene expression in TNF-α-induced** **FLS.** (**A**) Knockdown SMYD2 reduced H3K4me enrichment at A20’s promoter in 20 ng/ml TNF-α-induced FLS. After transfection with control siRNA or SMYD2 siRNA, then FLS were stimulated with 20 ng/ml TNF-α for 12 h, ChIP-PCR assay with an antibody against H3K4me. (**B**) Knockdown SMYD2 inhibited TNF-α-induced A20 expression. After transfection with control siRNA or SMYD2 siRNA, then FLS were stimulated with 20 ng/ml TNF-α for 12 h, mRNA expression of SMYD2 was detected. Data are presented as mean ± S.D, n=3, ^*^*p* < 0.05, ^***^*p* < 0.001.

**
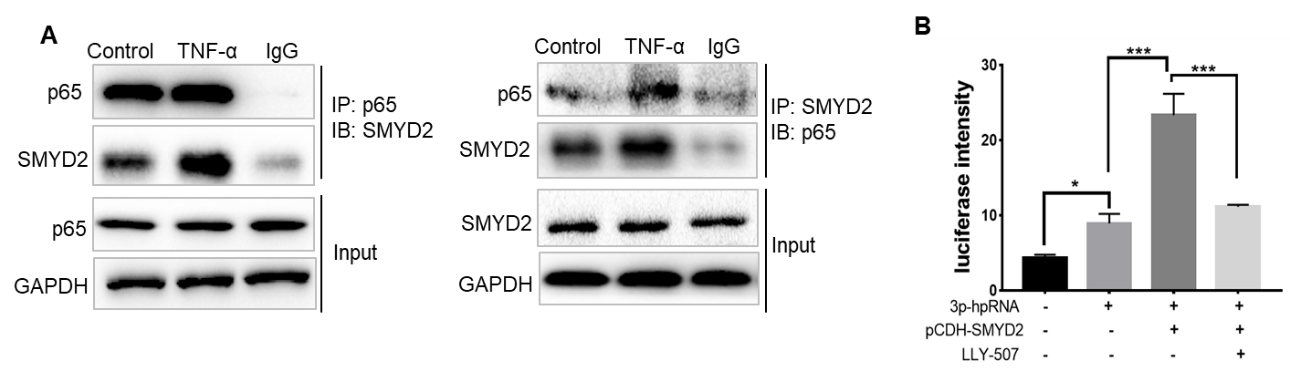
**

**Fig. S2 Co-IP of SMYD2 with p65 and SMYD2 contributes to IFN-β transcription activity. (A)** The synovial cells were treated with TNF-α for 1 h and cell lysates were subjected to immunoprecipitation using anti-p65 or SMYD2 antibody or IgG, the immunoprecipitate was separated by SDS-PAGE and blotted with p65 or SMYD2. **(B)** SMYD2 contributed to IFN-β transcription activity. Luciferase assay was performed to analyze IFN-β dependent transcriptional activity. Data are presented as mean ± S.D, n=3, **p* < 0.05, ****p* < 0.001.


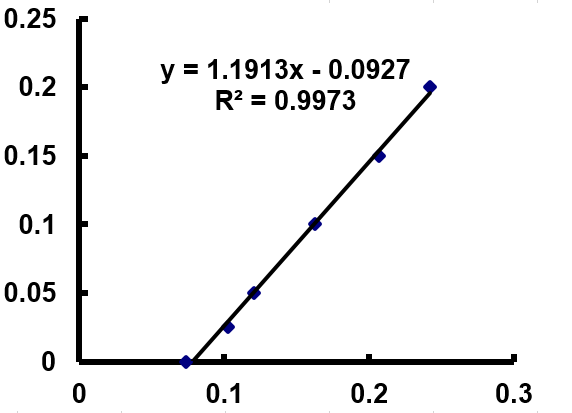


**Fig. S3 A significant linear positive correlation between TNB and initial AdoHcy concentration.** S-adenosyl-L-homocysteine (AdoHcy), a common product of methyltransferase, was hydrolyzed by S-adenosyl-L-homocysteine nucleosidase (SAHN) to form adenine and S-nucleoside homocysteine. The latter was further cleaved by S-ribosyl homocysteine (SRHH) to form homocysteine. Finally, homocysteine reacted with Ellman's reagent to form 5-thio-2-nitrobenzoic acid (TNB).

**
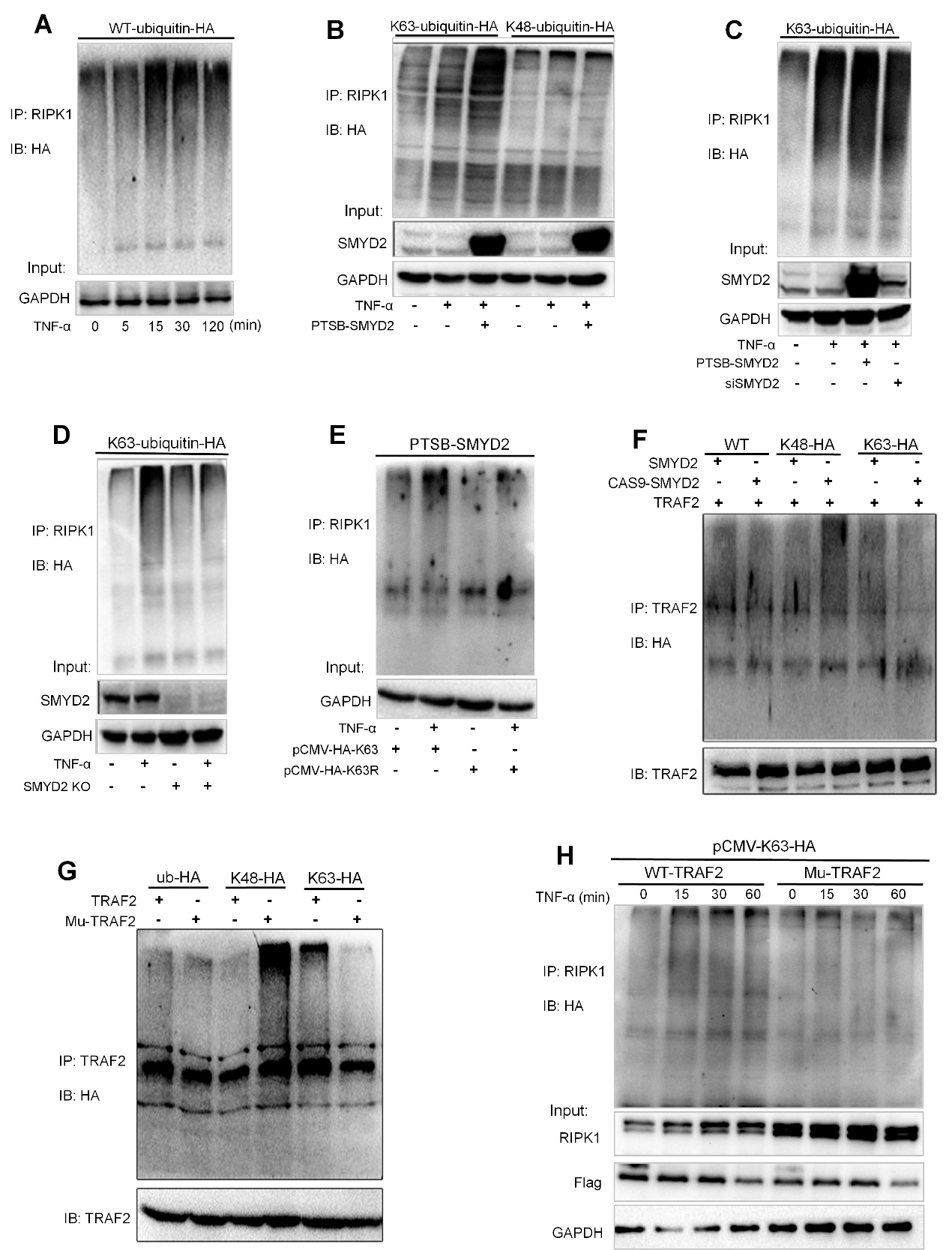
**

**Fig. S4 SMYD2 induces methylation mediates the k63 ubiquitin of RIPK1.** (**A**) TNF-α caused the ubiquitination of RIPK1 within 15 min in 293T cells transfected with HA-tagged ubiquitin. (**B**) Overexpression of SMYD2 significantly elevated the K63 chains modification on RIPK1 compared with the vehicle group stimulated with TNF-α, but had no effects on the K48-linked chains modification on RIPK1 in overexpressing SMYD2 or WT 293T cells transfected with pCMV-HA-K48 or pCMV-HA-K63 plasmids. (**C**) Knocking down of SMYD2 decreased the modification of K63 on RIPK1 in pCMV-HA-K63 293T cells transfected with SMYD2 siRNA or PTSB-SMYD2 plasmids. (**D**) Loss of SMYD2 could significantly decline the level of K63 chains on RIPK1 compared with the WT cells. Crispr-Cas9 system was applied in 293T cells to knockout the gene expression of *SMYD2*. (**E**) K63R mutant decreased RIPK1 ubiquitination level in overexpressed SMYD2 293T cells transfected with the K63 ubiquitin or point mutant K63R ubiquitin plasmids, which further mean SMYD2 specifically induces the k63 ubiquitin of RIPK1 (**F**) SMYD2 could also impede the degradation of TRAF2 and enhance its function by increasing K63 modification level. (**G**) K63 ubiquitination level was declined in mutant group and the mutant TRAF2 could be susceptible to K48 modification in pCMV-K63-HA, K48-HA, ub-HA 293T cells transfected with WT TRAF2 or mutant TRAF2 plasmids. (**H**) TRAF2 mutant decreased RIPK1 ubiquitination level in pCMV-K63-HA 293T cells transfected with WT TRAF2 or mutant TRAF2 plasmids.

**
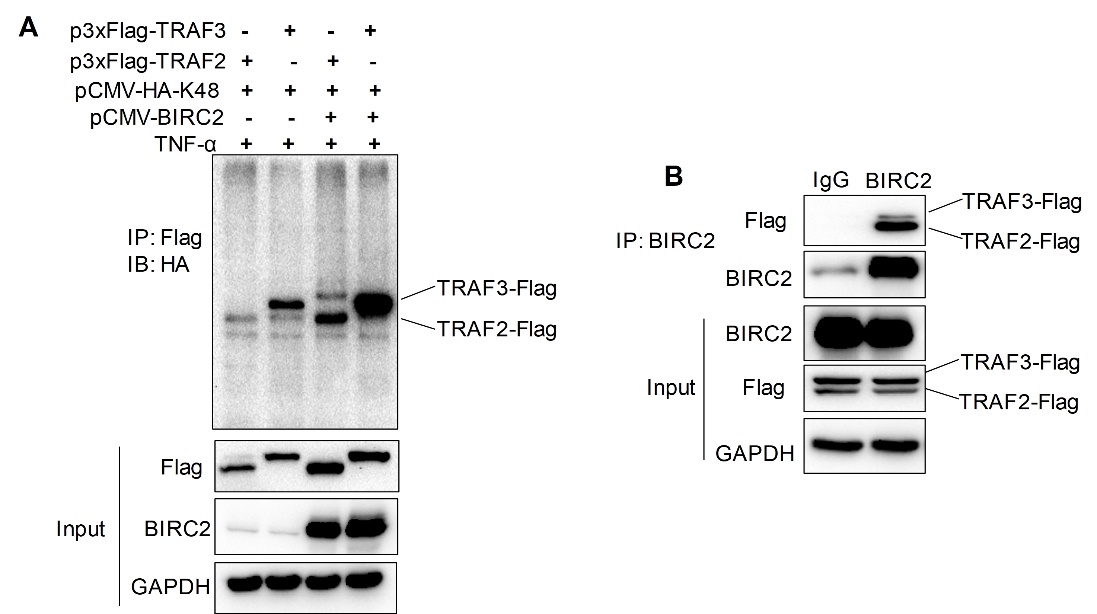
**

**Fig. S5 BICR2 possesses a strong affinity with TRAF2 for degradation than TRAF3.** pCMV-HA-K48 293T cells were transfected with p3xFlag-TRAF3, p3xFlag-TRAF2 or pCMV-BIRC2, along with TNF-α for 24 h and cell lysates were subjected to immunoprecipitation using Flag, the immunoprecipitate was separated by SDS-PAGE and blotted with HA (**A**); Cells lysates were subjected to immunoprecipitation using BIRC2, the immunoprecipitate was separated by SDS-PAGE and blotted with Flag (**B**).

**
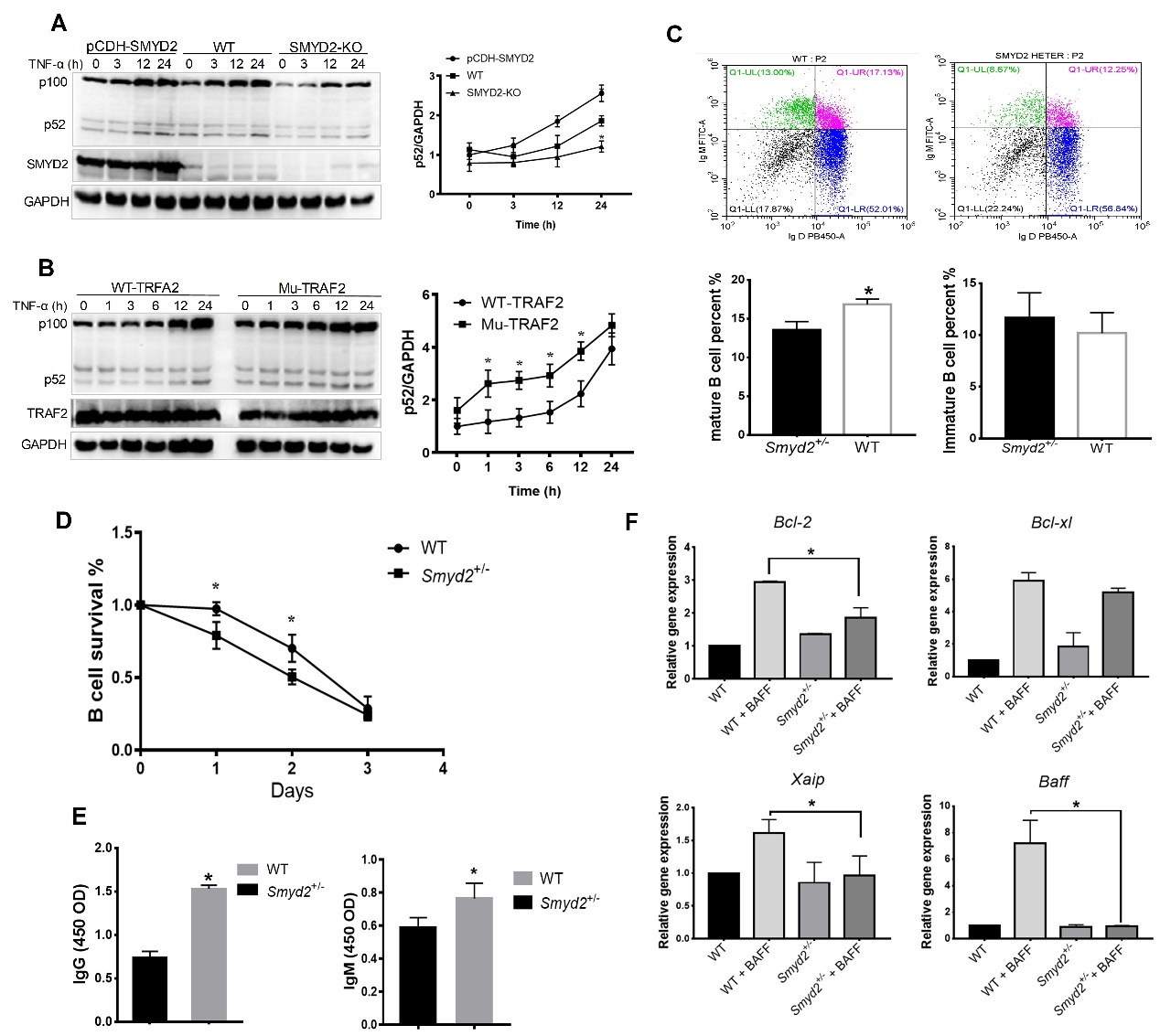
**

**Fig. S6 SMYD2 maintains mature B cells survival through the non-canonical NF-κB pathway.** (**A**) Knockout of SMYD2 alleviated the activation of p52. SMYD2-KO and SMYD2 overexpressed 293T cells were stimulated with TNF-α (20 ng/ml) for the indicated times, immunoblot analyzed for p100, p52, and SMYD2. (**B**) The mutant TRAF2 activates the non-canonical NF-κB pathway. Overexpressed 293T cells transfected with WT and the mutant TRAF2 were stimulated with TNF-α (20 ng/ml) for the indicated times, immunoblot analyzed for p100, p52, and TRAF2. (**C**) The number of mature B cells (CD19^+^ IgD^+^ IgM^+^) in the SMYD2^+/-^ mice was less than the WT mice, the immature B cells (CD19^+^ IgM^+^ IgD^-^) showed no difference in both groups. (**D**) The deficiency of SMYD2 decreased the viability of B cells. Purified the pan B cells from WT and SMYD2^+/-^ mice spleen and evaluated the survival capability without stimulation. (**E**) The concentrations of both the IgM and IgG in the serum from the SMYD2^+/-^ mice treated with DSS were lower from the WT mice treated with DSS. (**F**) The anti-apoptosis genes were reduced in the Smyd2^+/-^ mice. The pan B cells were collected from WT and SMYD2^+/-^ mice spleens and incubated with 50 ng/ml BAFF the ligand for BAFF/Blys receptor 3 overnight, the mRNA level of related survival genes *Bcl-2*, *Xaip* and *Baff* was detected by qPCR. Data are presented as mean ± S.D, n=5, **p* < 0.05.

**
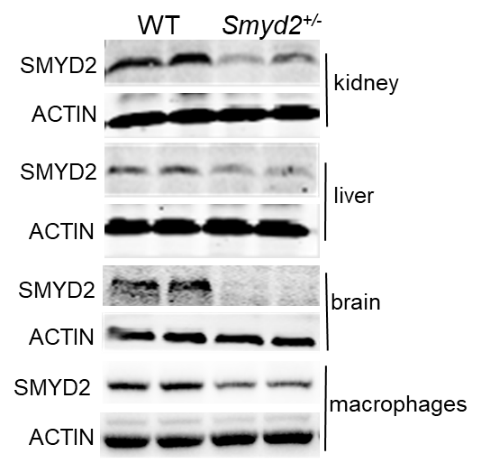
**

**Fig. S7 SMYD2 protein expression in main organs and macrophages of WT and *Smyd2*^+/-^ mice.** Immunoblot analysis of SMYD2 protein expression in kidney, liver, brain and macrophages of WT and *Smyd2*^+/-^ mice.

**
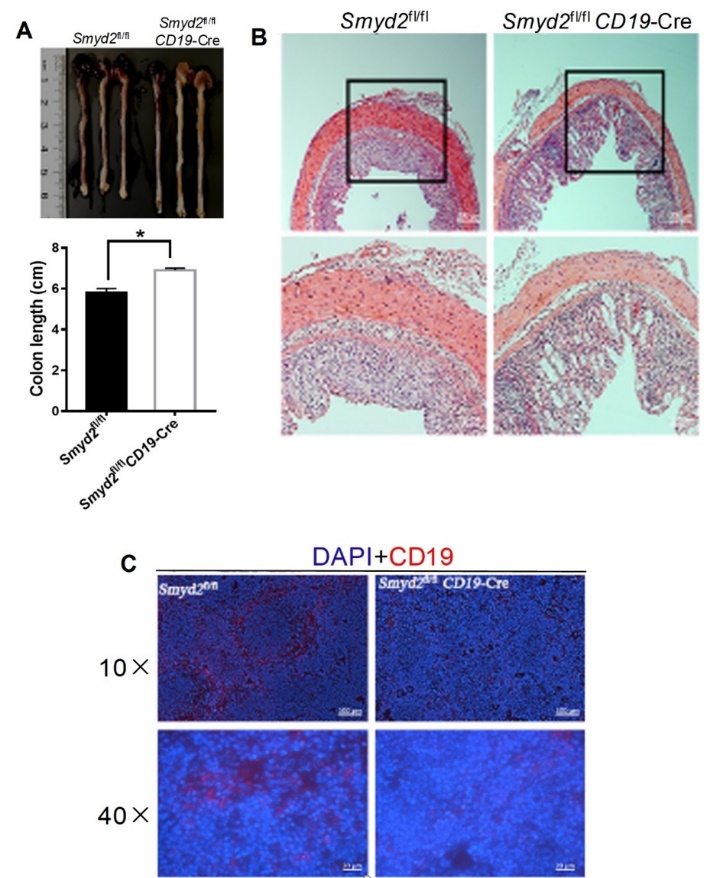
**

**Fig. S8 SMYD2 conditional knockout mice hinders the inflammation process in DSS colitis mice.** The *Smyd2*^fl/fl^ and *Smyd2*^fl/fl^ *CD19*-Cre mice with acute colitis induced by 3.5% (w/v) DSS for 7 days, the colon length in *Smyd2*^fl/fl^ and *Smyd2*^fl/fl^ *CD19*-Cre mice were detected. Data are presented as mean ± S.D, n=5, **p* < 0.05 **(A)**; HE staining of the colon tissues from *Smyd2*^fl/fl^ and *Smyd2*^fl/fl^ *CD19*-Cre mice **(B)**; the expression of the CD19 was observed in the spleen from *Smyd2*^fl/fl^ and *Smyd2*^fl/fl^ *CD19*-Cre mice **(C)**.


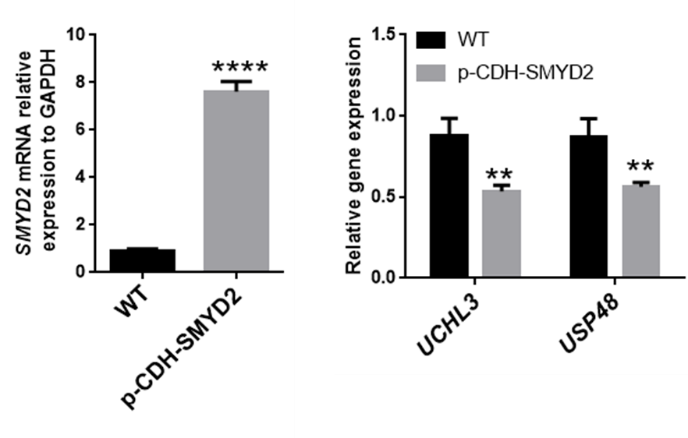


**Fig S9. SMYD2 overexpression decreased mRNA expression of UCHL3 and USP48.** *p*^****^ < 0.0001, *p*^**^ < 0.01 *vs*. WT.

**Table S1** Plasmids constructed were listed.

| Plasmid | Source | Identifier |
| --- | --- | --- |
| pCDH-SMYD2 | This paper | N/A |
| pTSB-CMV-SMYD2 | This paper | N/A |
| pGMNF-KB-Lu | YEASEN | 11501ES03 |
| pGL3-IFN-Beta | MiaoLingBio | P5314 |
| pRL-TK | PROMEGA | E2241 |
| pCMV-SMYD1 (C-Myc) | This paper | N/A |
| pCMV-SMYD2 (C- Myc) | This paper | N/A |
| pCMV-SMYD3 (C- Myc) | This paper | N/A |
| pCMV-SMYD4 (C- Myc) | This paper | N/A |
| pCMV-SMYD5 (C- Myc) | This paper | N/A |
| pCMV-MYND (C- Myc) | This paper | N/A |
| pCMV-SET (C- Myc) | This paper | N/A |
| pCMV-C-domain (C- Myc) | This paper | N/A |
| pCMV-SMYD2 (C-HA) | This paper | N/A |
| pCMV-SMYD2 (C-Flag) | This paper | N/A |
| pCMV-TRAF2 (C-His) (K115R-TRAF2) | This paper | N/A |
| pCMV-TRAF2(C-His) (K194R-TRAF2) | This paper | N/A |
| pCMV-TRAF2(C-His)(Double-mutation) | This paper | N/A |
| pCMV-TRAF2 (C-His) | This paper | N/A |
| pCMV-LSD1 (C-Myc) | This paper | N/A |
| pGEX-6P-1-SMYD2 | This paper | N/A |
| pCMV-HA-Ubiquitin | This paper | N/A |
| pCMV-HA-K48 | This paper | N/A |
| pCMV-HA-K63 | This paper | N/A |
| plko.1-shSMYD2 | This paper | N/A |
| pCMV-BIRC2 (C-His) | This paper | N/A |
| pCMV-A20 (C-His) | This paper | N/A |
| PX459-TRAF2 | This paper | N/A |
| PX459-SMYD2 | This paper | N/A |
| p3×Flag-TRAF2 | This paper | N/A |
| p3×Flag-TRAF3 | This paper | N/A |
| pTSB-CMV-TRAF2 | This paper | N/A |
| pTSB-CMV-TRAF2 (double-mutation) | This paper | N/A |

**Table S2** Primers used for qRT-PCR validation.

| Gene name | Primer name | Primer sequence (5´ to 3´) |
| --- | --- | --- |
| SMYD2 | Homo _SMYD2_F | TGAGTGCCAGGAGTGTACCA |
|  | Homo _SMYD2_R | CGTTGCGTGCATATCTGACC |
| GAPDH | Homo _GAPDH_F | GATTCCACCCATGGCAAATTCC |
|  | Homo _GAPDH_R | GCATCGCCCCACTTGATTTT |
| Bcl-2 | Mouse _Bcl-2_ F | ATGCCTTTGTGGAACTATATGGC |
|  | Mouse _Bcl-2_ R | GGTATGCACCCAGAGTGATGC |
| Bcl-xl | Mouse _Bcl-xl_ F | AGGCGATGAGTTTGAACTGC |
|  | Mouse _Bcl-xl_ R | TGAAGCTGGGATGTTAGATCACT |
| Xiapi | Mouse _Xiapi_ F | CGAGCTGGGTTTCTTTATACCG |
|  | Mouse _Xiapi_ R | GCAATTTGGGGATATTCTCCTGT |
| Baff | Mouse _Baff_ F | CAGCGACACGCCGACTATAC |
|  | Mouse _Baff_ R | CCTCCAAGGCATTTCCTCTTTT |
| GAPDH | Mouse _GAPDH_ F | CTTCTCTTGTGACAAAGTGGACAT |
|  | Mouse _GAPDH_ R | TTCTCAGCCTTGACTGTGCC |
